# Gut-derived Unpaired-3 cytokine signaling promotes systemic hypoxia tolerance in *Drosophila*

**DOI:** 10.1242/dmm.052999

**Published:** 2026-09-01

**Authors:** Kate Ding, Prajakta Bodkhe, Byoungchun Lee, W. Aline Ingelson-Filpula, Danielle Polan, Amy Wycislik, Tiffany Cheung, Sophie Wu, Reeha Rahim, Savraj S. Grewal

**Affiliations:** Department of Biochemistry and Molecular Biology, Arnie Charbonneau Cancer Institute, Alberta Children's Hospital Research Institute, Cumming School of Medicine, University of Calgary, Alberta T2N 4N1, Canada

**Keywords:** *Drosophila*, Hypoxia, Cytokine, Inter-organ signaling, Physiology, Adipose

## Abstract

Systemic hypoxia – a reduction in oxygen supply to all tissues and organs – occurs in many physiological and pathological conditions, including fetal development, high-altitude exposure, and disorders such as sleep apnea and respiratory disease. Under these conditions, whole-body physiology must adapt to ensure proper tissue functioning and survival. Although extensive research has characterized how individual cells sense and adapt to low-oxygen conditions, the mechanisms that coordinate whole-body responses to systemic hypoxia remain poorly understood. In this study, we uncovered an inter-organ signaling response mediated by the cytokine Unpaired-3 (Upd3), a functional homolog of human interleukin-6 (IL-6), that is important for systemic hypoxia tolerance in *Drosophila*. We demonstrated that hypoxia rapidly induces Upd3 expression and activates JAK/STAT signaling in larvae and adults. Interestingly, we discovered a sex-specific requirement for this pathway, with females, but not males, requiring Upd3 for hypoxia survival. We also identified the intestine as a critical source of hypoxia-induced Upd3 and showed that gut-derived Upd3 signals to the fat body and oenocytes to mediate hypoxia tolerance by promoting expression of nitric oxide synthase, the FGF ligand Branchless and the kinase Hipk. Furthermore, we revealed an unexpected role for the canonical hypoxia response transcription factor HIF-1α/Sima as a molecular brake, which prevents lethal Upd3 overproduction, revealing that hypoxia survival requires precise cytokine dosage control. Our findings define a gut-to-fat body signaling axis that coordinates systemic hypoxia adaptation, highlighting cytokine-mediated inter-organ communication as a mechanism for whole-body adaptation to low oxygen, with potential relevance to hypoxia-related human pathologies.

## Teaser

**Summary:** In *Drosophila*, the intestine releases the cytokine Upd3, which signals to the fat body and oenocytes to drive systemic hypoxia tolerance, a process kept in check by HIF-1α.

## INTRODUCTION

Oxygen is essential for aerobic life, serving as the terminal electron acceptor in cellular respiration that powers energy production across the animal kingdom. When oxygen availability becomes limited – a condition known as hypoxia – organisms must rapidly adapt their physiology and metabolism to maintain homeostasis (Semenza, 2011). These adaptations are critical not only during normal animal development and in specific ecological niches, but also in numerous pathological conditions that result in oxygen deprivation, such as stroke and lung disorders (Bickler and Buck, 2007; Jahani et al., 2020; Ramirez et al., 2007; Samanta et al., 2017; Semenza, 2014b; Stupnikov and Cardoso, 2017).

Although atmospheric air contains ∼20% oxygen, cells and tissues typically experience significantly lower oxygen levels under physiological conditions. For example, the early fetus develops in an environment in which oxygen levels can be as low as 1% (Dunwoodie, 2009), while oxygen levels in adult tissues can vary from 1% to 10% depending on the organ (McKeown, 2014). In many diseases, oxygen supply becomes further restricted, either locally (as in tumors, ischemia and stroke) or systemically [as in respiratory disorders, coronavirus disease 2019 (COVID-19) and sleep apnea] (May and Mehra, 2014; Semenza, 2011, 2014a,b; Xie and Simon, 2017). Systemic hypoxia results from insufficient oxygen reaching all the body's tissues and organs, potentially leading to multi-organ failure and life-threatening complications. Extensive work in cell culture has established how individual cells sense and respond to low oxygen, particularly how they modulate gene expression and reprogram metabolism to survive hypoxic conditions (Holdsworth and Gibbs, 2020; Nakazawa et al., 2016; Schito and Rey, 2018; Xie and Simon, 2017). In contrast, responses to systemic hypoxia in whole animals represent a fundamentally different challenge, requiring coordination across multiple tissues and organs (Baik and Jain, 2020; Midha et al., 2023; Samanta et al., 2017). These responses likely rely on inter-organ communication networks, but the nature of these networks and how they operate remain largely unexplored.

*Drosophila melanogaster* provides an excellent model system for investigating organismal adaptive responses to hypoxia (Callier et al., 2015, 2013; Farzin et al., 2014; Harrison et al., 2018; Harrison and Haddad, 2011). In their natural ecology, *Drosophila* larvae grow in rotting, fermenting food rich in microorganisms – an environment characterized by low ambient oxygen (Callier et al., 2015; Markow, 2015). They have, therefore, evolved multiple sophisticated mechanisms to tolerate hypoxia. One key pathway involves the conserved hypoxia-sensing transcription factor HIF-1α (known as Sima in *Drosophila*), which is stabilized under low-oxygen conditions and subsequently activates genes involved in metabolic adaptation and oxygen delivery (Centanin et al., 2008, 2005; Gorr et al., 2006; Lavista-Llanos et al., 2002; Romero et al., 2007; Texada et al., 2019). *Drosophila* research has also pioneered our understanding of inter-organ communication networks, revealing how specific tissues can detect environmental changes and signal to other organs to coordinate whole-body physiological responses (Alvarez-Ochoa et al., 2021; Boulan et al., 2015; Kannangara et al., 2021; Kim et al., 2021b; Leopold and Perrimon, 2007; Malita and Rewitz, 2021; Medina et al., 2022; Meschi and Delanoue, 2021; Okamoto and Watanabe, 2022; Owusu-Ansah and Perrimon, 2015; Rajan and Perrimon, 2011; Texada et al., 2020; Yoon et al., 2023). Thus, although all cells can adjust their metabolism in response to changes within their local environment, these tissue-level sensors ensure that physiological adaptations to environmental conditions are coordinated across multiple organs according to the organism's overall needs. This has been best exemplified in the context of how flies adapt to fluctuations in dietary nutrient availability. Extensive work has shown how tissues such as the gut, fat body and brain couple sensing of nutrients to behavioral, physiological and metabolic adaptations through a complex inter-organ signaling network (Droujinine and Perrimon, 2016; Koyama et al., 2020; Medina et al., 2022; Meschi and Delanoue, 2021; Tennessen and Thummel, 2011). A few reports have indicated that similar tissue-to-tissue signaling responses also underlie organismal adaptation to hypoxia. For example, Sima activation in the larval fat body triggers fat-to-brain signals that suppress insulin-like peptide production, reducing growth under oxygen-limited conditions (Texada et al., 2019), while Sima induction in specialized neurons modifies neuroendocrine signaling to stimulate blood cell production in hypoxia (Cho et al., 2018). Nevertheless, the inter-organ networks coordinating systemic hypoxia adaptation remain poorly understood.

The Unpaired (Upd) family of cytokines are key regulators of inter-organ communication in *Drosophila* (Zandawala and Gera, 2024). These secreted signaling molecules activate the conserved JAK/STAT pathway by binding to their cell surface receptor, Domeless (Agaisse and Perrimon, 2004). Upds play an important role in mediating communication between tissues to maintain metabolic and physiological homeostasis, especially in response to fluctuations in nutrient availability (Ingaramo et al., 2020; Rajan et al., 2017; Rajan and Perrimon, 2012; Zhao and Karpac, 2017). Additionally, Upds can be induced by a variety of stressors, including pathogenic infection, oxidative stress, nutrient stress and tissue damage (Hersperger et al., 2024; Srinivasan et al., 2016; Yang et al., 2015). Importantly, the source of Upd production varies with the specific stress context, as does the target tissue and the resulting physiological effects. This context-dependent signaling allows Upds to orchestrate complex, multi-tissue responses that coordinate activities across the gut, fat body, brain, cardiac tissue and immune cells to control tissue repair, metabolism, endocrine signaling and growth (Brent and Rajan, 2020; Cai et al., 2021; Chakrabarti et al., 2016; Ding et al., 2021; Gera et al., 2022; Hersperger et al., 2024; Houtz et al., 2017; Huang et al., 2020; Ingaramo et al., 2020; Jiang et al., 2009; Kierdorf et al., 2020; Liu et al., 2025, 2024; Nagai et al., 2023; Obata et al., 2018; Osman et al., 2012; Rai et al., 2025; Rajan et al., 2017; Rajan and Perrimon, 2012; Romao et al., 2021; Shin et al., 2020; Sodders et al., 2025; Srinivasan et al., 2016; von Frieling et al., 2020; Wang et al., 2014; Woodcock et al., 2015; Yang et al., 2015; Zhao and Karpac, 2017; Zhou et al., 2013).

A critical aspect of Upd signaling is its context-dependent role in stress responses, which can be either adaptive or detrimental to organismal fitness. In acute stress conditions, Upd induction often promotes beneficial protective mechanisms. For instance, transient activation of the Upds in gut epithelial cells following injury or infection stimulates stem cell-mediated tissue regeneration (Buchon et al., 2009; Jiang et al., 2009; Obata et al., 2018; Osman et al., 2012; von Frieling et al., 2020; Zhou et al., 2013). Similarly, injury-induced Upd3 from hemocytes supports tissue repair and survival (Chakrabarti et al., 2016). Additionally, in normal physiology, Upd3 production from pericardial cells maintains extracellular matrix (ECM) protein expression essential for cardiac function (Gera et al., 2022). However, these same signaling pathways become harmful when chronically activated or in pathological contexts. For example, persistent Upd activation in the aging intestine leads to epithelial dysplasia and shortened lifespan (Li et al., 2016). Upon high-fat diet consumption, hemocyte-derived Upd3 disrupts glucose homeostasis and reduces longevity (Woodcock et al., 2015), while fat body-derived Upd2 causes nephrocyte dysfunction (Zhao et al., 2025). Additionally, age-related Upd3 upregulation in oenocytes contributes to cardiac dysfunction (Huang et al., 2020). Perhaps most strikingly, in tumor models, Upd3 secretion triggers systemic pathologies including blood–brain barrier compromise and metabolic dysregulation that accelerate mortality (Ding et al., 2021; Kim et al., 2021a; Liu et al., 2025). This dual nature – beneficial in physiological contexts but potentially harmful when dysregulated – underscores the importance of investigating Upd signaling in specific physiological and pathological conditions to understand its precise roles in stress adaptation.

Here, we investigate how Upd3 signaling mediates whole-body hypoxia tolerance in *Drosophila*, revealing an interorgan communication network that coordinates systemic hypoxia adaptation through the interplay of cytokine and classic hypoxia response pathways.

## RESULTS

### Hypoxia induces Upd3/JAK/STAT signaling

**Fig. 1.**
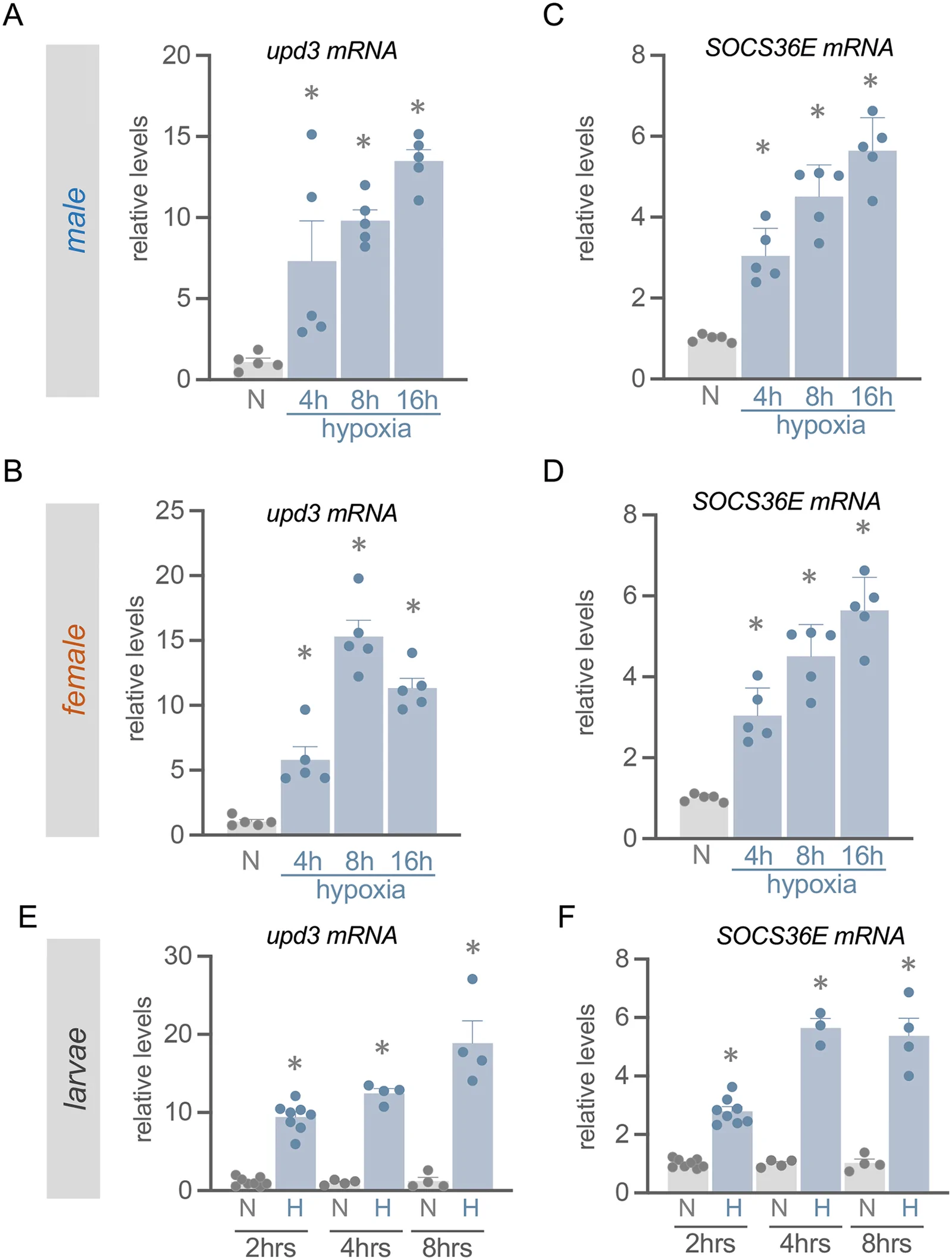
**Hypoxia induces Upd3/JAK/STAT signaling in larvae and adults.** (A-D) Age-matched flies (*w^1118^*) were maintained in normoxia or hypoxia (1% oxygen), then collected for gene expression analysis. Quantitative reverse transcription PCR (qRT-PCR) measurement of *upd3* in males (A) and females (B) following 4, 8 and 16 h of hypoxia. STAT-target gene *Socs36E* levels in males (C) and females (D) were measured following 4, 8 and 16 h of hypoxia. All qRT-PCR data were normalized to reference genes listed in Table S2. Data represent mean+s.e.m., *N*=5 independent groups of samples (five animals per group) per experimental condition. Data points represent independent samples. *P<0.05 compared to normoxia controls, one-way ANOVA followed by Dunnett's test. (E,F) qRT-PCR measurements of *upd3* mRNA (E) and *Socs36E* mRNA (F) in larvae exposed to 2, 4 and 8 h of hypoxia (1% oxygen). All qRT-PCR data were normalized to reference genes listed in Table S2. Data represent mean+s.e.m., *N*=3-8 independent groups of animals (ten animals per group) per experimental condition. Data points represent independent samples. *P<0.05, compared to normoxia controls, one-way ANOVA followed by Dunnett's test.

We first examined whether hypoxia induces the cytokine/JAK/STAT signaling pathway. To do this, we subjected *w^1118^* adult flies and larvae to different durations of either hypoxia (1% oxygen) or normoxia (ambient air) and then collected samples for gene expression analysis by quantitative reverse transcription PCR (qRT-PCR). We found that hypoxia exposure led to a strong upregulation of *upd3* mRNA levels in male and female adult flies (Fig. 1A,B), an effect that was apparent within 4 h of hypoxia exposure. To explore whether this upregulation led to functional induction of cytokine signaling, we also measured the mRNA levels of well-described STAT target genes as a functional readout of JAK/STAT pathway activation. This analysis revealed strong upregulation of the STAT target gene *Socs36E* in males and females with a similar time course (Fig. 1C,D). We also saw that another class of STAT regulated genes, the Turandot genes – *TotA*, *TotC* and *TotM* – were also induced upon hypoxia exposure in adults (Fig. S1A,B). Previous studies have shown that flies become immobilized and cease feeding during exposure to 1% oxygen. However, nutrient starvation for a 16-h period failed to induce *upd3* mRNA in both male (Fig. S1C) and female (Fig. S1D) animals, suggesting that cessation of feeding is not responsible for *upd3* induction in hypoxia. Finally, we examined whether the hypoxia induction of Upd/JAK/STAT signaling also occurred in larvae. We exposed larvae to 2, 4 and 8 h of normoxia or hypoxia and collected them for gene expression analysis. Similarly to our observations in adults, we detected induction of both *upd3* and *Socs36E* mRNA in hypoxic larvae (Fig. 1E,F). Together, these results indicate that induction of Upd3 cytokine signaling is a robust response to hypoxia exposure in *Drosophila.*

### Sex-specific requirement for Upd3 in hypoxia tolerance

**Fig. 2.**
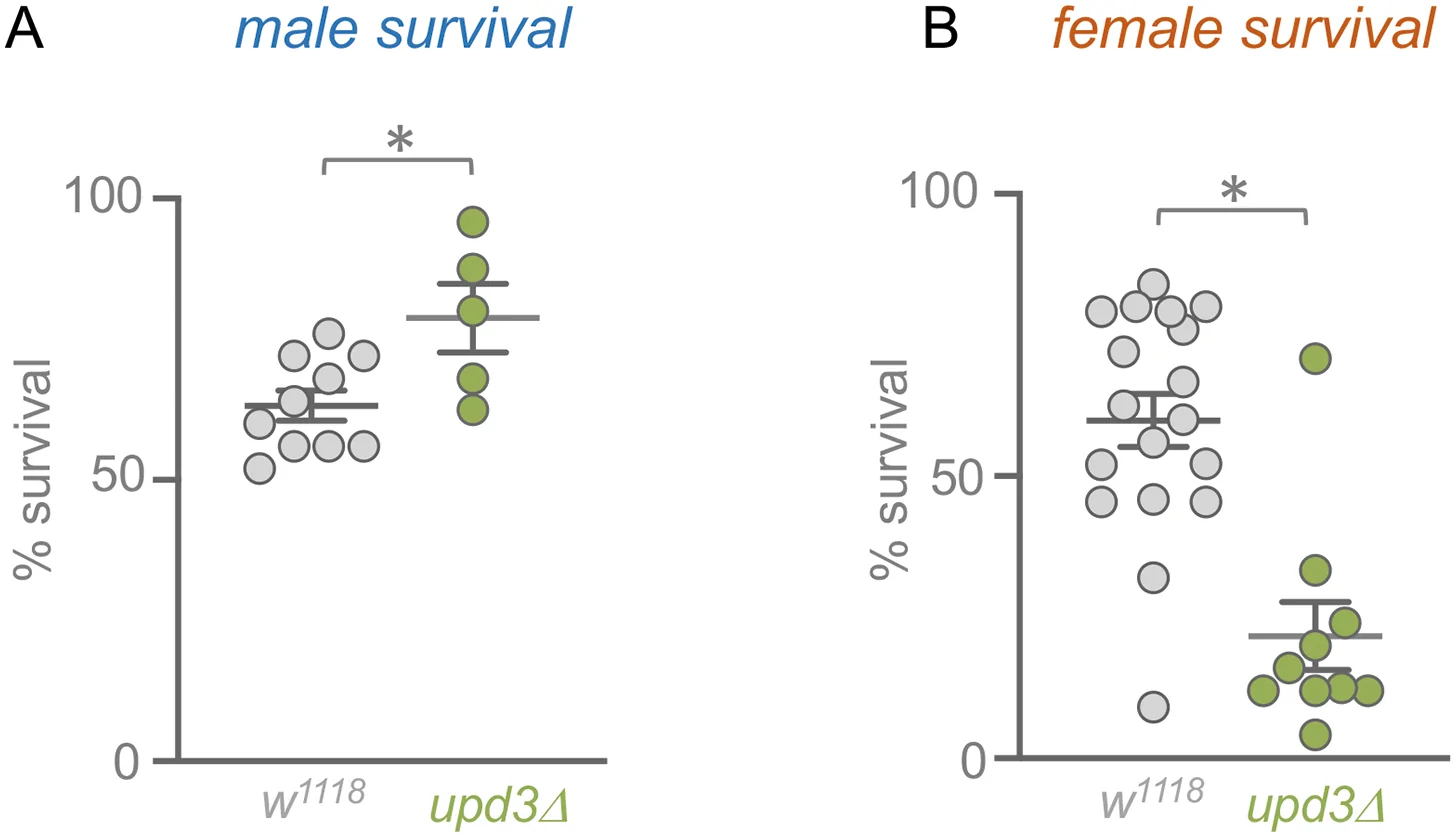
**Upd3 is required for hypoxia tolerance in females but not males.** (A,B) Hypoxia survival measured in control (*w^1118^*) versus *upd3*-null (*upd3*Δ) males (A) and females (B). Data represent mean±s.e.m., *N*≥5 independent groups of animals (15-25 per group) per experimental condition. *P<0.05, unpaired two-tailed *t*-test. Data points represent independent samples.

We next investigated the functional relevance of increased Upd3 signaling in hypoxia tolerance. To test this, we examined survival under hypoxic conditions in an *upd3*-null mutant line (*upd3Δ*) (Osman et al., 2012). We exposed age-matched 7-day-old control (*w^1118^*) and *upd3* mutant adult flies to an acute bout of hypoxia that resulted in 50-80% survival of control flies. Our survival analysis revealed that female *upd3*-null mutants exhibited significantly reduced survival compared to their control counterparts (Fig. 2B). In contrast, *upd3*-null mutant males showed no decrease in hypoxia survival compared to control males and even displayed a small, but significant, increase in survival (Fig. 2A). Given that female metabolism is altered by mating status (Ahmed et al., 2020; Hadjieconomou et al., 2020; Hudry et al., 2016; Reiff et al., 2015; Zipper et al., 2020), we asked whether mating status affected the requirement for *upd3* in hypoxia tolerance. Both mated and virgin female *upd3*-null animals showed decreased survival in hypoxia compared to controls (Fig. S2A,B).

These results suggest a sexually dimorphic requirement for *upd3* in mediating hypoxia tolerance. Although *upd3* is necessary for hypoxia tolerance in females regardless of mating status, it appears to be dispensable or even detrimental in males. Further studies will be needed to determine whether this sex difference occurs through the sex determination pathway, which is emerging as an important mediator of fly physiology (Mank and Rideout, 2021). However, for the remainder of this study, we focused on exploring how Upd3 mediates hypoxia tolerance in mated females.

### Intestinal Upd3 coordinates systemic hypoxia responses

The Upd cytokines facilitate tissue-to-tissue communication to control adaptive responses to stress, acting in an autocrine or paracrine manner from multiple tissue sources. One key tissue known to produce Upd3 in response to stress is the adult midgut, in which a variety of stresses – including infection, tissue damage and nutrient deprivation – can upregulate intestinal Upd3 to mediate local and systemic adaptive responses (Buchon et al., 2009; Cai et al., 2021; Houtz et al., 2017; Jiang et al., 2009; Nagai et al., 2023; Obata et al., 2018; Takeishi et al., 2013; von Frieling et al., 2020; Zhou et al., 2013). We therefore investigated whether the gut might similarly serve as a source of Upd3 to promote hypoxia tolerance.

**Fig. 3.**
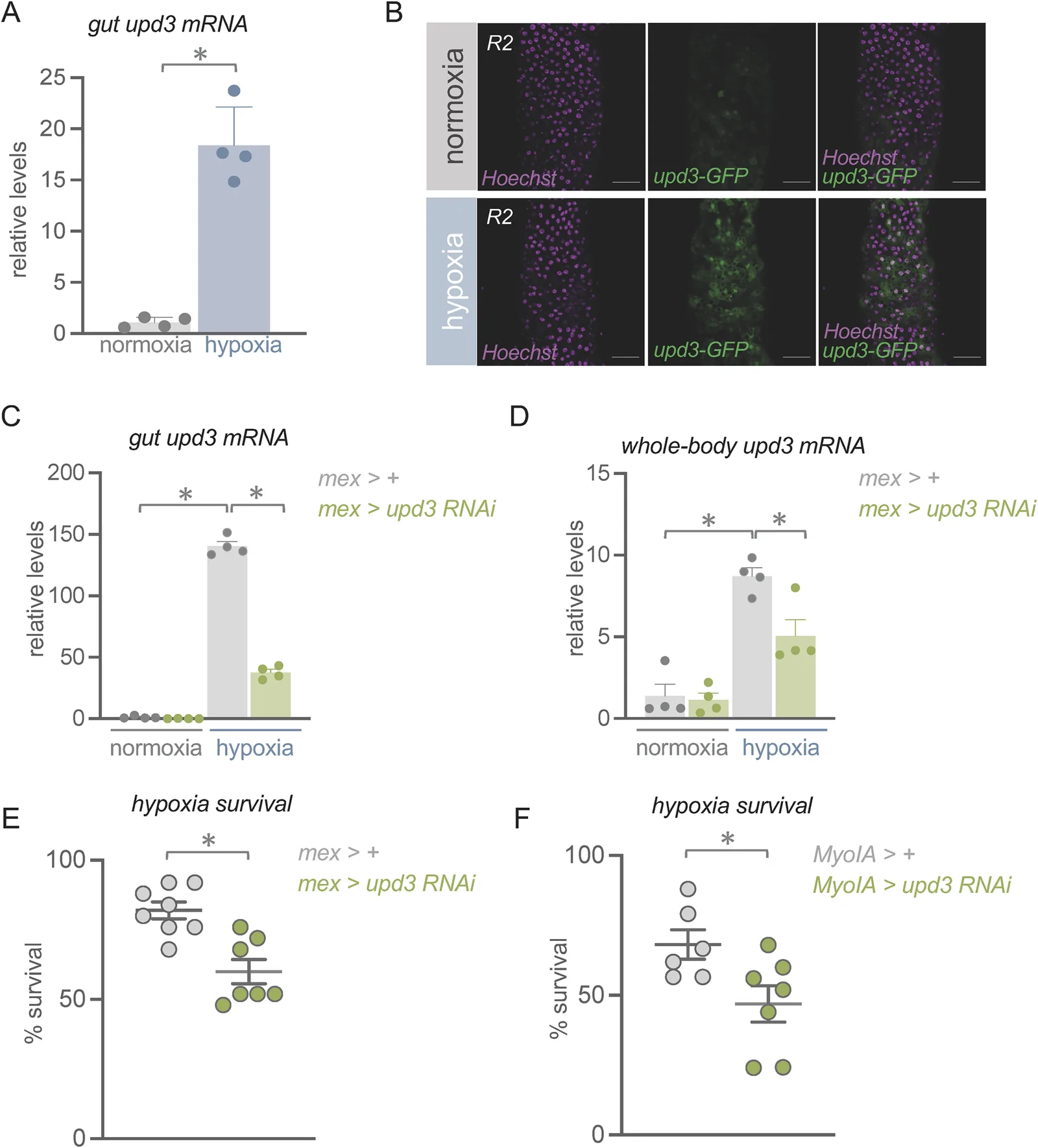
**Gut-derived Upd3 promotes hypoxia tolerance.** (A) qRT-PCR analysis of *upd3* mRNA of guts from *w^1118^* adult females maintained in normoxia or exposed to 16 h of hypoxia (1% oxygen). RNA was isolated from whole guts. All qRT-PCR data were normalized to reference genes listed in Table S2. Data represent mean+s.e.m., *N*=4 independent groups of samples (five intestines per group) per experimental condition. Data points represent independent samples. (B) Female flies expressing GFP under control of a *upd3* promoter (*upd3-Gal4, UAS-GFP*) were exposed to hypoxia (1% oxygen) or maintained in normoxia for 16 h. Whole guts were dissected, stained with Hoechst to visualize nuclei, and mounted for visualization of GFP expression. Representative images of R2 region shown. Scale bars: 50 μm. (C) qRT-PCR measurement of *upd3* mRNA in whole intestines of control (*mex>+*) or enterocyte Upd3 knockdown flies (*mex>upd3-RNAi*) in normoxia and hypoxia. All qRT-PCR data were normalized to reference genes listed in Table S2. Data represent mean+s.e.m., *N*=4 independent groups of samples (five intestines per group) per experimental condition. *P<0.05, Student's *t*-test following two-way ANOVA. Data points represent independent samples. (D) *upd3* mRNA also measured in whole body. All qRT-PCR data were normalized to reference genes listed in Table S2. Data represent mean+s.e.m., *N*=4 independent groups of samples (five animals per group) per experimental condition. *P<0.05, Student's *t*-test following two-way ANOVA. Data points represent independent samples. (E,F) Hypoxia survival measured in enterocyte Upd3 knockdown animals using two different drivers [*mex>upd3-RNAi* (E) and *Myo1A>upd3-RNAi* (F)] compared to their respective control genotypes (*mex>+, Myo1A>+*). Data represent mean±s.e.m., *N*≥6 independent groups of animals (15-25 per group) per experimental condition. *P<0.05, unpaired two-tailed *t*-test. Data points represent individual samples.

When we exposed control (*w^1118^*) females to 16 h of normoxia or hypoxia and dissected guts for qRT-PCR analysis, we found a strong upregulation of *upd3* mRNA levels in hypoxia (Fig. 3A). We also analyzed flies carrying a *upd3* GFP transcriptional reporter (*upd3-Gal4, UAS-GFP*) that were exposed to either normoxia or hypoxia. Microscopic visualization of the intestines from these flies showed that hypoxia-exposed animals had stronger GFP expression in their guts than did normoxia controls, which was especially evident in the R2 region of the intestine (Fig. 3B). Moreover, this increased GFP expression was mostly seen in the large epithelial enterocytes. To explore this finding further, we knocked down *upd3* using drivers for the two main intestinal cell types known to express *upd3* (Houtz et al., 2017): an enterocyte-specific driver (*mex-Gal4*), and a progenitor cell driver targeting stem cells and enteroblasts (*esg-Gal4*). We measured intestinal *upd3* mRNA levels by qRT-PCR and found that enterocyte-specific knockdown resulted in a significant reduction of ∼80% in *upd3* mRNA levels (Fig. 3C), whereas progenitor-specific knockdown produced a more modest reduction (Fig. S2C). These results indicate that enterocytes are the predominant intestinal cell type upregulating *upd3* in hypoxia, with a smaller contribution from progenitor cells. Furthermore, when we measured whole-body *upd3* mRNA levels in enterocyte-specific knockdown flies (*mex>upd3-RNAi*), we observed a significant ∼40% reduction in the hypoxia-induced upregulation of whole-body *upd3* (Fig. 3D), indicating that intestinal enterocytes account for a substantial proportion of the systemic *upd3* response to hypoxia.

We next investigated the functional role for enterocyte-derived Upd3 in hypoxia tolerance. We subjected control flies (*mex>+*) and flies with gut-specific *upd3* knockdown (*mex>upd3-RNAi*) to hypoxia and measured their survival. We saw that suppressing intestinal *upd3* expression significantly reduced fly survival compared to that of controls (Fig. 3E). We repeated this experiment using another enterocyte-specific driver, *Myo1A-Gal4*, to knock down *upd3* and observed similar results, with the *Myo1A>upd3-RNAi* flies showing significantly reduced survival compared to controls (*Myo1A>+*) (Fig. 3F). To rule out potential developmental effects of Upd3 knockdown, we employed a temperature-sensitive enterocyte driver to induce RNA interference (RNAi) knockdown of *upd3* specifically during adulthood. These adult-specific Upd3 knockdown flies (*Myo1A^ts^>upd3-RNAi*) also showed reduced survival under hypoxic conditions compared to controls (*Myo1A^ts^>+*) (Fig. S2D). We also saw no difference in survival between control (*mex>+*) and mCherry RNAi (*mex>mCherry-RNAi*) animals, showing that RNAi induction in the gut itself does not affect hypoxia tolerance (Fig. S2E). Collectively, these results demonstrate that the gut serves as an important source of hypoxia-induced *upd3* expression essential for hypoxia tolerance.

We also assessed *upd3* expression in isolated head (enriched in brain), thorax (enriched in muscle) and abdominal tissues (guts and ovaries removed, enriched in fat body and oenocytes) from normoxic and hypoxic flies, and found that *upd3* mRNA was induced by hypoxia in all three regions (Fig. S3A). We then knocked down *upd3* using drivers targeting the major cell types in each region – including neurons (*nSyb-Gal4*), muscle (*dMef2-Gal4*) and fat body/oenocytes (*Desat;r4-Gal4*) – and measured hypoxia survival. In contrast to gut-specific knockdown, none of these manipulations significantly affected survival compared to that of controls (Fig. S3B-D).

### Gut-derived Upd3 signals to fat and oenocytes to promote hypoxia tolerance

**Fig. 4.**
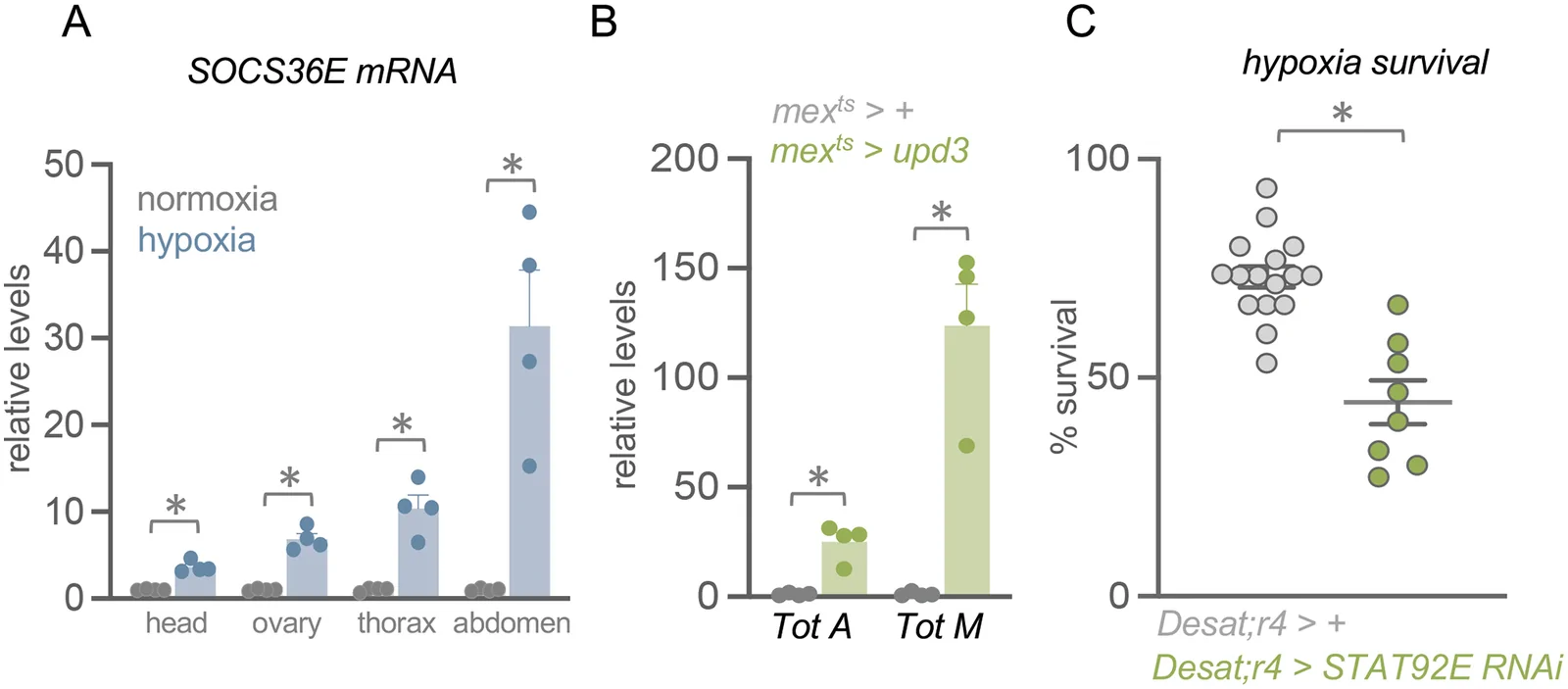
**Gut-to-abdominal tissue Upd3 signaling mediates hypoxia tolerance.** (A) Adult females (*w^1118^*) were maintained in normoxia or exposed to 16 h of hypoxia. Tissues including the head, ovary, thorax and abdomen were dissected for *Socs36E* mRNA expression analysis. All qRT-PCR data were normalized to reference genes listed in Table S2. Data represent mean+s.e.m., *N*=4 independent groups of samples (five to ten tissues per group) per experimental condition. *P<0.05, unpaired two-tailed *t*-test. Bars represent mean+s.e.m. Data points represent independent samples. (B) qRT-PCR analysis of STAT target genes (*TotA* and *TotM*) in the adult female abdominal tissue with intestinal Upd3 overexpression (*mex^ts^>upd3*) versus those in control (*mex^ts^>+*). All qRT-PCR data were normalized to reference genes listed in Table S2. Data represent mean+s.e.m., *N*=4 independent groups of samples (five abdomens with intestines and ovaries removed per group) per experimental condition. *P<0.05; Student's *t*-test following two-way ANOVA. Bars represent mean+s.e.m. Data points represent independent samples. (C) Survival measured in animals with RNA interference (RNAi) knockdown of Stat92E in the two main abdominal tissue types – oenocytes and fat body [*Desat;r4>Stat92E-RNAi* versus control (*Desat;r4>+*)]. Data represent mean±s.e.m., *N*≥8 independent groups of animals (15-25 per group) per experimental condition. *P<0.05, unpaired two-tailed *t*-test. Data points represent individual samples.

Having established the intestine as an important source of expression for Upd3 in mediating hypoxia tolerance, we next investigated which tissue(s) gut-derived Upd3 might be communicating with to stimulate JAK/STAT signaling and mediate this hypoxia tolerance response. We performed qRT-PCR on tissue from isolated heads, thoraces, abdomens (with guts and ovaries removed) and ovaries from female *w^1118^* flies exposed to either normoxia or hypoxia to examine expression of the STAT target gene, *Socs36E*. We saw that *Socs36E* was upregulated in all tissues in hypoxia-exposed flies, suggesting widespread stimulation of JAK/STAT signaling in low oxygen. However, the hypoxia induction of *Socs36E* was most pronounced in isolated abdominal tissues (Fig. 4A). We also saw strong induction of two other STAT target genes, *TotA* and *TotM*, in abdominal tissues of hypoxia-exposed flies (Fig. S4A). We then examined whether gut-derived Upd3 can signal to abdominal tissues, by inducing acute *upd3* expression from intestinal enterocytes (*mex^ts^>UAS-upd3*) and measuring expression of STAT target genes in isolated abdominal tissues. We observed induction of both *TotA* and *TotM* mRNA in the abdomens of *mex^ts^>UAS-upd3* compared to in the abdomens of controls (*mex^ts^>+*) (Fig. 4B), indicating that gut-derived Upd3 can stimulate JAK/STAT signaling in abdominal tissues.

Adult abdominal tissue is composed mostly of fat body and oenocytes (Ghosh et al., 2020). These tissues are functionally equivalent to mammalian adipose and liver and play important roles in lipid and sugar storage, mobilization and metabolism, as well as function as endocrine tissues (Arrese and Soulages, 2010; Bland, 2022; Ghosh et al., 2020; Huang et al., 2022; Kuhnlein, 2011; Martínez et al., 2020; Meschi and Delanoue, 2021; Sriskanthadevan-Pirahas et al., 2022; Stefana et al., 2017; Yamada et al., 2018). Through these effects, the fat body and oenocytes both have well-established roles in coordinating whole-body physiological responses to changes in environmental stimuli, such as food, pathogens and toxins. In particular, previous studies have shown that the fat body is a key target of endocrine Upd3 signaling involved in the regulation of systemic physiology and stress responses (Gera et al., 2022; Liu et al., 2025). We therefore assessed the functional importance of abdominal STAT signaling in hypoxia tolerance. We used a line carrying two independent Gal4 drivers, *Desat-Gal4* and *r4-Gal4*, that drive expression specifically in oenocytes and fat body, respectively. We used this line to express dsRNA against Stat92E (*Desat;r4>Stat92E-RNAi*) simultaneously in both oenocytes and fat body and measured hypoxia survival. We found that animals with oenocyte/fat body-specific Stat92E knockdown showed significantly reduced survival under hypoxic conditions compared to control animals (Fig. 4C). We also saw no difference in survival between control (*Desat;r4>+*) or GFP RNAi (*Desat;r4>GFP-RNAi*) animals, indicating that RNAi induction in the fat body or oenocytes itself has no effect on hypoxia tolerance (Fig. S4B). Together, these results establish fat body and oenocytes as key target tissues through which gut-derived Upd3 promotes hypoxia tolerance.

### Gut-derived Upd3 controls abdominal expression of *nitric oxide synthase*, *branchless* and *Hipk* – three regulators of hypoxia survival

We next explored potential fat body/oenocyte targets of gut-derived Upd3 in the regulation of hypoxia tolerance, focusing on previously described regulators of hypoxia responses in *Drosophila*. Nitric oxide (NO), which is synthesized by the enzyme Nitric oxide synthase (Nos), is a conserved mediator of hypoxic responses and is required for several aspects of hypoxia adaptation in *Drosophila*, including cell cycle arrest, altered behavior and survival (DiGregorio et al., 2001; Dijkers and O'Farrell, 2009; Teodoro and O'Farrell, 2003; Wingrove and O'Farrell, 1999). Branchless (Bnl) is the fly homolog of FGF with roles in hypoxia-induced trachea formation and the control of fat body metabolism (Centanin et al., 2008; Newton et al., 2020). Hipk is a kinase that we previously showed promotes hypoxia tolerance (Ding et al., 2022).

**Fig. 5.**
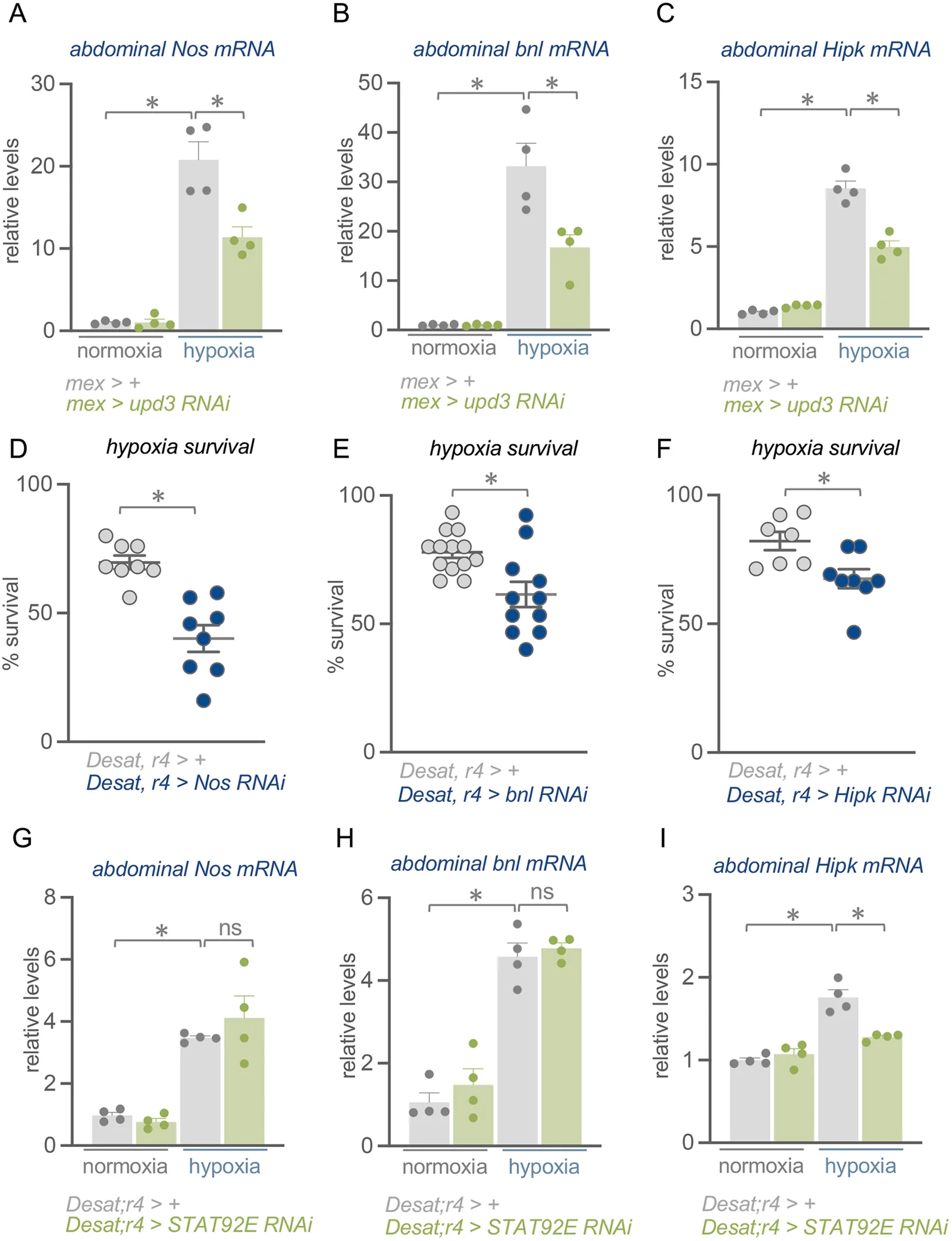
**Gut-derived Upd3 controls fat/oenocyte expression of the hypoxia regulators Nos, Bnl and Hipk.** (A-C) qRT-PCR measurement of mRNA levels of *Nos* (A), *bnl* (B) and *Hipk* (C), from abdominal tissues of control flies (*mex>+*) versus those of flies with intestinal enterocyte Upd3 knockdown (*mex>upd3-RNAi*). *N*=4 independent groups of samples (each sample contains abdomens of four flies with intestines and ovaries removed) per experimental condition. All qRT-PCR data were normalized to reference genes listed in Table S2. *P<0.05, Student's *t*-test following two-way ANOVA. Bars represent mean+s.e.m. Data points represent independent samples. (D-F) Hypoxia survival measured in animals with combined fat body and oenocyte knockdown of *Nos* (*Desat;r4*>*Nos*-RNAi; D), *bnl* (*Desat;r4*>*bnl*-RNAi; E) or *Hipk* (*Desat;r4*>*Hipk*-RNAi; F) compared to that in controls (*Desat;r4*>+). Data represent mean±s.e.m., *N*≥4 independent groups of animals (15-25 per group) per experimental condition. *P<0.05, unpaired two-tailed *t*-test. Data points represent individual samples. (G-I) qRT-PCR measurement of *Nos* mRNA levels (G), *bnl* mRNA levels (H) and *Hipk* mRNA levels (I), from abdominal tissues of control flies (*Desat;r4>+*) versus those in flies with combined fat body and oenocyte *Stat92E* knockdown (*Desat;r4>Stat92E RNAi*). *N*=4 independent groups of samples (each sample contains abdomens of four flies with intestines and ovaries removed) per experimental condition. All qRT-PCR data were normalized to reference genes listed in Table S2. *P<0.05; ns, not significant; Student's *t*-test following two-way ANOVA. Bars represent mean+s.e.m. Data points represent independent samples.

We found that mRNA expression levels of all three regulators were upregulated in isolated abdominal tissues from hypoxia-exposed flies. To test whether these genes are targets of gut-derived Upd3, we knocked down *upd3* specifically in intestinal enterocytes (*mex>upd3-RNAi*) and measured expression of all three genes in abdominal tissues from normoxic and hypoxic flies. Gut-specific *upd3* knockdown significantly reduced the hypoxia-induced expression of *Nos*, *bnl* and *Hipk* in abdominal tissues (Fig. 5A-C), indicating that gut-derived Upd3 is required for their induction.

To determine whether these genes are functionally required for hypoxia tolerance, we used the combined *Desat;r4-Gal4* driver to knock down each gene specifically in fat body and oenocytes. In all three cases, knockdown significantly reduced survival under hypoxic conditions compared to that in controls (Fig. 5D-F), demonstrating that *Nos*, *bnl* and *Hipk* are each required in fat body and oenocytes for hypoxia tolerance.

Together, these results identify *Nos*, *bnl* and *Hipk* as downstream effectors of gut-derived Upd3 signaling in the control of hypoxia tolerance. To explore whether Upd3 controls their expression through direct JAK/STAT signaling in fat body and oenocytes, we knocked down *Stat92E* specifically in these tissues using the *Desat;r4-Gal4* driver and measured expression of all three genes. Interestingly, fat body/oenocyte-specific loss of *Stat92E* suppressed hypoxia-induced *Hipk* expression but did not affect *Nos* or *bnl* expression (Fig. 5G-I). These results suggest that gut-derived Upd3 controls expression of these effectors through both direct (*Hipk*) and indirect (*Nos* and *bnl*) relay mechanisms.

### HIF-1α limits Upd3 signaling to promote hypoxia tolerance

**Fig. 6.**
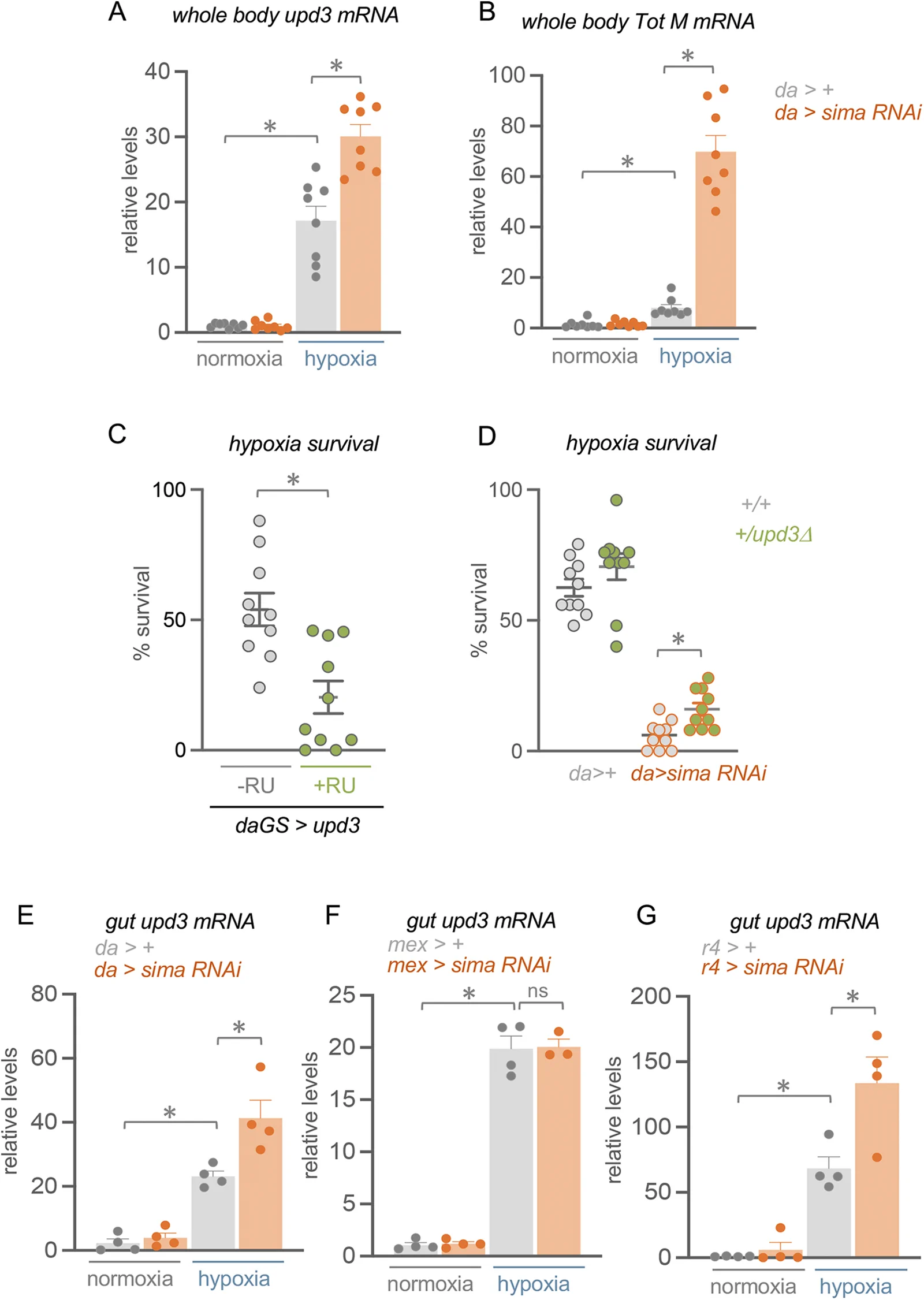
**HIF-1α/Sima limits excess Upd3/JAK/STAT signaling.** (A,B) Control (*da>+*) or Sima knockdown (*da>sima-RNAi*) females were maintained in normoxia or exposed to hypoxia (1% oxygen) for 16 h. Whole flies were then collected for qRT-PCR analysis. Whole-body *upd3* mRNA (A) and *TotM* mRNA (B) were measured. All qRT-PCR data were normalized to reference genes listed in Table S2. Data represent mean+s.e.m., *N*=8 independent groups of samples (five animals per group) per experimental condition. *P<0.05; Student's *t*-test following two-way ANOVA. Data points represent independent samples. (C) Hypoxia survival of control (*da-GS>upd3*, −RU486) versus ubiquitous *upd3* overexpression (*da-GS>upd3*, +RU486) adult flies. Data represent mean±s.e.m., *N*=10 independent groups of animals (15-25 per group) per experimental condition. *P<0.05, unpaired two-tailed *t*-test. Data points represent individual samples. (D) Control *(da>+)*, *upd3*Δ heterozygote (*da>upd3*Δ/+), *sima* knockdown (*da>sima-RNAi*) and *upd3*Δ heterozygote *sima* knockdown (*da>upd3*Δ/+*;sima-RNAi*) adult females were maintained in normoxia or exposed to hypoxia (1% oxygen), and survival was measured. Data represent mean±s.e.m., *N*≥8 independent groups of animals (15-25 per group) per experimental condition. *P<0.05, unpaired two-tailed *t*-test. Data points represent individual samples. (E-G) Gut *upd3* mRNA levels, assayed by qRT-PCR, in *da>+* versus *da>sima-RNAi* flies (ubiquitous *sima* knockdown; E), *mex>+* versus *mex>sima-RNAi* flies (enterocyte-specific *sima* knockdown; F) and *r4>+* versus *r4>sima-RNAi* flies (fat-body-specific *sima* knockdown; G). Flies were maintained in normoxia or hypoxia (1% oxygen) for 16 h, and whole guts were immediately dissected for qRT-PCR analysis. All qRT-PCR data were normalized to reference genes listed in Table S2. Data represent mean+s.e.m., *N*=4 independent groups of samples (five intestines per group) per experimental condition. *P<0.05; ns, not significant; Student's *t*-test following two-way ANOVA. Data points represent independent samples.

We next explored how hypoxia regulates Upd3 expression. Hypoxia-inducible factor 1 (HIF-1) is one of the best-characterized transcriptional regulators of hypoxia response, and, in mammalian cells, has been shown to be pro-inflammatory, in part through expression of cytokines (Nizet and Johnson, 2009; Palazon et al., 2014; Palsson-McDermott et al., 2015; Tannahill et al., 2013). We therefore investigated whether HIF-1α (known as Sima in *Drosophila*) is required for Upd3 induction during hypoxia. We used RNAi to knock down Sima ubiquitously using the *da-Gal4* driver (*da>sima-RNAi*) and compared *upd3* mRNA levels under hypoxic conditions to those in controls (*da>+*). Interestingly, we found that Sima knockdown augmented the hypoxia-induced increase in *upd3* mRNA levels (Fig. 6A) and led to a strong amplification of the hypoxia-induced increase in expression of the STAT target gene, *TotM* (Fig. 6B). These results suggest that, rather than being required for Upd3 induction, Sima is needed to limit Upd3/JAK/STAT signaling during hypoxia. This finding aligns with the prevailing immunological principle that cytokine signaling must be precisely fine-tuned. Although effective immune responses rely on rapid induction of cytokine signaling, it is equally important to limit excess cytokine signaling to avoid ‘cytokine-storm’ responses, whereby unchecked cytokine signaling can cause unwanted tissue damage and immunopathology (Cron et al., 2023; Fajgenbaum and June, 2020; Jahani et al., 2020; Medzhitov, 2021; Meizlish et al., 2021; Ye and Medzhitov, 2019). Based on our results, we hypothesized that one role of Sima during hypoxia is to limit the potentially deleterious effects of excessive Upd3 levels to ensure proper adaptation to hypoxia. This hypothesis made two testable predictions: first, overexpression of Upd3 to create excess cytokine signaling would be detrimental in hypoxia; and second, if the role of Sima is to dampen excessive Upd3 signaling, then lowering Upd3 levels might partially protect against the lethality seen with Sima knockdown. We tested both predictions.

To test whether excess Upd3 is detrimental in low oxygen, we used the GeneSwitch-Gal4 system to acutely overexpress *upd3* ubiquitously in adult female flies. Upd3-overexpressing flies (*daGS>upd3*, fed RU486) showed significantly reduced hypoxia survival compared to control flies (*daGS>upd3*, fed vehicle control) (Fig. 6C). We confirmed this result using another ubiquitous inducible driver (*actGS-Gal4*) and observed the same outcome – excess *upd3* reduced hypoxia tolerance (Fig. S5A).

We next tested whether reducing *upd3* gene dosage could rescue the lethality of Sima knockdown flies. To lower *upd3* signaling, we carried out experiments in flies heterozygous for *upd3*. Using qRT-PCR, we first verified that the augmented *upd3* levels seen in hypoxic Sima knockdown flies were reduced back to control hypoxic levels in *upd3* heterozygotes, demonstrating that reducing *upd3* gene dosage is sufficient to restore *upd3* levels to the normal hypoxic range (Fig. S5B). We then tested hypoxia survival and found that Sima knockdown flies heterozygous for the *upd3* deletion (*upd3Δ*/+*; da>sima-RNAi*) showed a small, but significant, improvement in survival under hypoxic conditions compared to that of Sima knockdown flies with normal *upd3* levels (*da>sima-RNAi*) (Fig. 6D). The modest nature of this rescue is expected, given that HIF-1/Sima coordinates many gene expression changes required for hypoxia tolerance, and loss of Sima likely disrupts multiple essential processes that may not be restored by reducing *upd3* dosage alone. Nevertheless, these results implicate excessive Upd3 signaling as one small, but significant, contributing factor to the lethality observed in *sima*-deficient flies, consistent with Sima functioning to limit cytokine levels during hypoxia.

### Adipose HIF-1α regulates gut Upd3 expression

Our experiments on Sima focused on whole-body regulation of Upd3, so we next turned to how Sima might regulate intestinal Upd3 production specifically. We repeated the experiment with ubiquitous Sima knockdown but measured gut *upd3* mRNA levels directly. As with whole-body Upd3, we observed augmented Upd3 production in the gut under hypoxic conditions (Fig. 6E), suggesting that the modulation of whole-body Upd3 levels by Sima largely reflects augmentation of Upd3 in the gut.

We initially hypothesized that Sima in the gut directly antagonizes gut Upd3 expression. To test this hypothesis, we generated enterocyte-specific Sima knockdown flies and isolated their intestines after exposure to normoxia or hypoxia to measure *upd3* mRNA levels. Contrary to our expectation, gut-specific Sima knockdown (*mex>sima-RNAi*) did not significantly alter intestinal *upd3* mRNA levels compared to those in controls (*mex>+*) under hypoxic conditions (Fig. 6F). This finding suggested that gut Sima does not directly regulate gut Upd3 expression and that the effect of Sima on Upd3 is non-autonomous.

Previous work has shown that Sima activity in the fat body during hypoxia plays an important non-autonomous role in controlling whole-body physiology, with effects thought to rely on Sima modulation of fat-dependent endocrine signaling (Texada et al., 2019). The *Drosophila* fat body also serves as a central integration hub for signals to and from other tissues. Based on these observations, we examined the fat body as a potential source of Sima-dependent regulation of intestinal Upd3. We generated fat body-specific *sima* knockdown flies (*r4>sima-RNAi*), exposed them to hypoxia and measured gut *upd3* mRNA levels. Strikingly, intestines from fat body *sima* knockdown flies showed significantly greater induction of Upd3 under hypoxic conditions compared to that in control (*r4>+*) intestines (Fig. 6G). These results suggest that Sima in the fat body acts as a hypoxia sensor that regulates Upd3 expression in the gut, implying the existence of a Sima-dependent signal mediating this tissue-to-tissue communication.

We investigated whether reactive oxygen species (ROS) might mediate this inter-organ communication. Because ROS are well-established inducers of Upd3, we tested whether Sima knockdown affects ROS-induced Upd3 by exposing fat body-specific Sima knockdown flies (*r4>sima-RNAi*) to paraquat, an ROS-generating chemical. We observed no significant difference in intestinal Upd3 expression compared to that in controls (Fig. S6A). We also overexpressed the antioxidant genes *CatA*, *Sod1* or *Sod2* individually in the fat body or enterocytes and measured hypoxia-induced *upd3* expression. In all conditions tested, hypoxia-induced *upd3* expression was unaffected (Fig. S6B-G). These results suggested that hypoxia- and Sima-dependent modulation of Upd3 is independent of ROS signaling.

## DISCUSSION

Organisms must coordinate whole-body responses to environmental stresses through inter-organ communication networks. Our results reveal how cytokine signaling mediates such coordination during systemic hypoxia, identifying a gut-to-fat body axis that is essential for organismal survival under low-oxygen conditions.

Upd3 has been shown to mediate adaptive responses to numerous environmental stresses, including nutrient stress, infection and toxins, and our work extends its role to the regulation of adaptation to low oxygen. We demonstrate that gut-derived Upd3 is needed for survival in low oxygen, and our results suggest that this occurs in part through signaling to two main tissues in the fly abdomen – the oenocytes and the fat body. Both cell types play important roles in energy metabolism, particularly in coordinating whole-body metabolic and physiological adaptations to changes in environmental conditions through their adipose- and liver-like roles in fat and sugar storage and mobilization, as well as their endocrine functions in sensing nutrient changes and signaling to other tissues (Arrese and Soulages, 2010; Bland, 2022; Ghosh et al., 2020; Huang et al., 2022; Kuhnlein, 2011; Martínez et al., 2020; Meschi and Delanoue, 2021; Sriskanthadevan-Pirahas et al., 2022; Stefana et al., 2017; Yamada et al., 2018). Although we see that *upd3* is induced in multiple tissues during hypoxia, tissue-specific knockdown in neurons, muscle and fat body/oenocytes did not significantly affect hypoxia survival, suggesting that gut-derived Upd3 makes the predominant contribution to hypoxia tolerance. We cannot exclude, however, that *upd3* produced in other tissues contributes to additional aspects of hypoxia adaptation, either independently or in combination with gut-derived Upd3.

Our results show that gut-derived Upd3 controls expression of *Nos*, *bnl* and *Hipk* in fat body and oenocytes, and that all three are required for hypoxia tolerance. Interestingly, our data suggest that Upd3 regulates these effectors through direct and indirect mechanisms. Fat body/oenocyte-specific loss of JAK/STAT signaling suppressed *Hipk* expression but not *Nos* or *bnl*, suggesting that although *Hipk* is a direct transcriptional target of JAK/STAT signaling in these tissues, *Nos* and *bnl* are controlled through relay mechanisms that do not require JAK/STAT activation within fat body and oenocytes themselves. There is precedent for such indirect relay effects in the context of other Upd-mediated inter-organ responses. For example, in response to CO_2_, neuronal Upd3 controls blood cell differentiation in the lymph gland not directly but through an intermediate step in which Upd3 first signals to the fat body to induce Dilp6 (also known as Ilp6), which then signals to the lymph gland (Cho et al., 2018). Similarly, upon infection, gut-derived Upd2 controls olfactory behavior via a relay in which Upd2 signals to glial cells, which in turn alter apolipoprotein expression to modify olfactory neuron function (Cai et al., 2021). Our results suggest that analogous relay mechanisms may operate downstream of gut-derived Upd3 in the control of fat body and oenocyte gene expression.

The precise mechanisms through which Nos, Bnl and Hipk promote hypoxia tolerance remain to be defined, but their previously described roles suggest several intriguing possibilities. NO, produced downstream of Nos, acts as a second messenger through activation of cGMP and PKG signaling, and NO/cGMP/PKG signaling has been shown to mediate physiological and behavioral responses to hypoxia in *Drosophila*, including by controlling gene expression, tracheogenesis and the cell cycle (DiGregorio et al., 2001; Dijkers and O'Farrell, 2009; Teodoro and O'Farrell, 2003; Wingrove and O'Farrell, 1999). NO can also mediate post-translational protein modifications through nitrosylation, with a recent study showing that nitrosylation can enhance the unfolded protein response (Cho et al., 2024), a conserved pathway induced by hypoxia (Wouters and Koritzinsky, 2008). Bnl is the *Drosophila* FGF homolog, with well-described roles in hypoxia-induced tracheal remodeling (Centanin et al., 2008), and has also been shown to regulate fat body metabolism and function (Newton et al., 2020), which may be important for metabolic adaptation to low oxygen. Hipk is a kinase that can modulate multiple signaling pathways (Blaquiere et al., 2014; Chen and Verheyen, 2012; Lee et al., 2009a,b; Tettweiler et al., 2019; Verheyen et al., 2012) and has been shown in flies to control glucose metabolism and mitochondrial function (Wong et al., 2020, 2019), which may be relevant to hypoxia adaptation. Together, these effectors suggest that gut-derived Upd3 coordinates a broad downstream response in fat body and oenocytes. When Upd3 signaling is lost, the failure to induce these effectors likely compromises multiple aspects of the hypoxia adaptive response simultaneously, which may underlie the increased vulnerability of *upd3* loss animals compared to that of controls. Defining the full spectrum of gene expression changes induced by gut-derived Upd3 in these tissues will be an important avenue for future work and is likely to uncover additional effectors and pathways through which Upd3 cytokine signaling promotes hypoxia tolerance.

Our findings support a model in which systemic responses to hypoxia, like those to other environmental stresses such as starvation and infection, rely on tissue-specific stress sensors and tissue-to-tissue coordination. Thus, although all cells can sense hypoxia and mount adaptive cell-autonomous responses, specific tissues also trigger non-autonomous signals that coordinate whole-body physiological adaptation. In *Drosophila*, previous examples include Sima activation in the larval fat body, triggering fat-to-brain signals that suppress Insulin-like peptide production under oxygen-limited conditions (Texada et al., 2019), and Sima induction in specialized neurons modifying neuroendocrine signaling to stimulate blood cell production (Cho et al., 2018). Similar principles operate in mammals, in which specialized tissues detect oxygen levels and trigger systemic responses – for example, oxygen sensing in keratinocytes of the epidermal layer of the skin through HIF-1α, and mitochondrial metabolism triggers EPO production from the kidney to promote red blood cell production in the bone marrow (Boutin et al., 2008; Hamanaka et al., 2016), while carotid body sensing triggers neural and endocrine responses that modify respiration and circulation (Stupnikov and Cardoso, 2017). The physiology and anatomy of the fly intestine make it an ideal oxygen sensor. It is extensively innervated by a dense network of trachea that provide oxygen to support its functions and maintain its structure (Blackie et al., 2024; Li et al., 2013; Perochon et al., 2021; Tamamouna et al., 2021). This extensive tracheal network might also enable the gut to sense changes in environmental oxygen and coordinate whole-body responses via its central role as an endocrine organ. Our findings expand the view of the gut as a multipurpose environmental sensor that can detect diverse stimuli including pathogens, nutrients and oxygen availability to coordinate systemic adaptations. Interestingly, studies in the mosquito *Aedes aegypti* have shown that gut hypoxia acts as a systemic signal, activating HIF to coordinate fat body metabolism and whole-body growth (Valzania et al., 2018), supporting the notion of the gut being a hypoxia sensor that controls whole-body physiology through effects on the fat body.

We observed a striking sexual dimorphism in this cytokine hypoxia response – although both sexes show upregulated Upd3/JAK/STAT signaling in hypoxia, females require Upd3 for hypoxia tolerance whereas males do not. Notably, sex-specific effects of Upd cytokines have been reported in other contexts, including tumor growth and intestinal metabolism (Hudry et al., 2019; Wang et al., 2024), suggesting that sexual dimorphism in Upd signaling is a broader phenomenon. The effects we observe are independent of mating status, as both virgin and mated females require Upd3. Studies have established that the sex determination pathway in flies can drive male–female differences in many aspects of physiology and metabolism, with whole-body metabolic differences often conferred by the sexual identity of specific cells and tissues that control and establish organismal metabolic state (Belmonte et al., 2019; Hudry et al., 2019, 2016; Mank and Rideout, 2021; Millington et al., 2021a,b; Pomatto et al., 2017; Regan et al., 2016, 2022; Rideout et al., 2015; Sawala and Gould, 2017; Wat et al., 2020, 2021). Moreover, these male–female differences in physiology confer sexually dimorphic sensitivity to environmental stresses such as oxidative stress and starvation (Pomatto et al., 2017; Wat et al., 2020). Understanding how sex determination pathways might regulate Upd3 signaling would be an interesting avenue to explore.

An intriguing aspect of our findings is the unexpected role of HIF-1α in restraining cytokine signaling during hypoxia. Mammalian HIF-1α is typically pro-inflammatory through control of interleukin production in immune cells (Nizet and Johnson, 2009; Palazon et al., 2014; Palsson-McDermott et al., 2015; Tannahill et al., 2013). However, our results suggest a more nuanced model in which Sima/HIF-1α can serve dual roles: activating essential hypoxia-response genes, while simultaneously preventing excessive cytokine responses that could compromise survival. The concept of restraining cytokine signaling is well established in immunology, where too little or too much signaling can each prove deleterious. Our work suggests that HIF-1α may function as part of this regulatory network, ensuring that hypoxia-induced cytokine responses remain within beneficial ranges. The tissue-specific nature of this regulation – with fat body Sima controlling intestinal cytokine production – indicates that Sima operates within a distributed regulatory system that coordinates appropriate stress responses across different organs. The molecular mechanisms underlying this inter-tissue communication remain to be determined but may involve Sima-dependent regulation of secreted factors that signal from fat body to gut. Interestingly, Sima has previously been shown to antagonize innate immune signaling through other pathways (Bandarra et al., 2015), suggesting that restraining inflammatory responses may be a broader function of HIF-1α, depending on the specific stress conditions. Our work also points to what might be an important feature of Upd biology – Upd3 levels must be carefully balanced to support hypoxia tolerance. Our results show that too little Upd3 compromises female survival, whereas excessive levels are equally detrimental. The small but significant partial rescue of Sima knockdown lethality by reducing Upd3 dosage is consistent with excessive Upd3 contributing to this phenotype, although the modest nature of this effect likely reflects the fact that Sima coordinates many processes required for hypoxia tolerance beyond cytokine regulation. More broadly, extensive work has shown that Upds can be beneficial or harmful depending on context, and our work suggests that the strength of the signaling response may be a critical factor in determining whether outcomes are adaptive or pathological in hypoxia.

One open question is how hypoxia controls Upd3 expression in the gut – both the direct induction by hypoxia and the non-autonomous modulation by fat body Sima. We found that the induction is independent of ROS, a well-established pathway for Upd3 induction under stress conditions. The induction is also independent of gut Sima, contrasting with mammalian systems in which HIF-1α increases similar cytokines such as IL-6. The *upd3* locus can integrate an array of different signaling inputs to induce gene expression in response to enteric pathogen infection (Houtz et al., 2017), and the hypoxia response may rely on one or more of these pathways. Metabolic changes in enterocytes could also be important. For example, dietary methionine has been shown to control Upd expression through modulation of S-adenylmethionine metabolism (Obata et al., 2018). Interestingly, recent work has shown that, in larvae, disruption of glycolysis leads to defects in larval growth and development mediated through upregulation of Upd3 (Rai et al., 2025). Given the critical role of Sima in maintaining glycolysis (Heidarian et al., 2025), the elevated gut Upd3 we observe when blocking Sima in the fat body may arise due to disrupted glycolysis and potentially metabolic signaling from fat body to gut.

Our findings may have important implications for understanding systemic hypoxia responses in mammals. The Upd3 homolog in mammals is IL-6, a classic stress-induced inflammatory cytokine (Qing et al., 2020). Heightened IL-6 responses are observed in many disorders associated with systemic hypoxia in humans – such as sleep apnea, chronic obstructive pulmonary disease and COVID-19 – and are thought to mediate inflammatory responses and contribute to pathology (Ayres, 2020; de Lima et al., 2016; Nadeem et al., 2013). However, our work raises the possibility that IL-6 could also be beneficial in mediating adaptive responses under physiological hypoxia. Just as we find that Upd3 is essential for adaptive hypoxia tolerance in flies, IL-6 may serve important adaptive and homeostatic functions in mammals under conditions of systemic hypoxia.

## MATERIALS AND METHODS

### Fly husbandry

*Drosophila* stocks were maintained at 25°C or 18°C. All stocks were maintained on medium composed of 100 g *Drosophila* Type II agar, 1200 g cornmeal, 490 mg Torula yeast, 450 g sugar, 1240 g D-glucose and 160 ml acid mixture (propionic and phosphoric acid) in 20 l water. Genetic crosses were established by mating virgin females with males. All crosses and progeny were maintained at 25°C. The following fly stocks were used in this study: *upd3*Δ [Bloomington *Drosophila* Stock Center (BDSC) 55728], *UAS-upd3* (gifted from Bruno Lemaitre, École Polytechnique Fédérale de Lausanne, Lausanne, Switzerland), *UAS-upd3 RNAi* [Vienna *Drosophila* Resource Center (VDRC) 27136/GD], *UAS-sima RNAi* (VDRC 106187/KK), *UAS-Nos RNAi* (BDSC 57700), *UAS-bnl RNAi* (VDRC 101377), *UAS-Hipk RNAi* (BDSC 35363), *UAS-Stat92E RNAi* (VDRC 43866/GD), *UAS-CatA* (BDSC 24621), *UAS-Sod1* (BDSC 33605), *UAS-Sod2* (BDSC 24494), *upd3-Gal4, UAS-GFP* (gifted from Erica Bach, NYU Grossman School of Medicine, New York, NY, USA), *mex-Gal4ts* (gifted from Bruce Edgar, Huntsman Cancer Institute, University of Utah, Salt Lake City, UT, USA), *esg-Gal4* (gifted from Bruce Edgar), *da-Gal4* (BDSC 8641), *r4-Gal4* (BDSC 33832), *Desat-Gal4* (BDSC 65404), *mex-Gal4* (BDSC 91368), *Myo1A-Gal4* (gifted from Bruce Edgar), *Myo1A-Gal4ts* (Jiang et al., 2009), *dMef2-Gal4* (BDSC 25756), *nSyb-Gal4* (BDSC 51941), *UAS-GFP RNAi* (BDSC 9330), *UAS-mCherry RNAi* (BDSC 35785), *da*-*Gal4 GeneSwitch* (Sun et al., 2014) and *Act-Gal4 GeneSwitch* (Sun et al., 2014). We used FlyBase (FB2026_01) to find information on all genes and fly stocks used in this study (Jenkins et al., 2022; Öztürk-Çolak et al., 2024). See Table S1 for complete list of lines used.

### Hypoxia exposure

For all hypoxia experiments, *Drosophila* were placed into an airtight chamber in standard food vials with a constant flow of premixed 1% oxygen (1% oxygen/99% nitrogen) at room temperature. The flow rate was controlled using an Aalborg model P gas flow meter.

### Adult hypoxia survival

The duration of hypoxia exposure was tailored to each experiment to produce 50-80% survival in control animals. Males show reduced hypoxia tolerance compared to females. Accordingly, females were exposed for 24-28 h and males for 16-18 h (in groups of 15-25 per vial) depending on the specific experiment. The number of flies that were viable 24 h after the hypoxia exposure period were then counted.

### RU486 treatment

Mated females were placed in vials (15-25 per vial) containing standard *Drosophila* medium supplemented with 100 μM RU486 in ethanol or equal volume of ethanol alone as vehicle control for 5-7 days.

### Starvation

7-day-old adult flies were placed in vials (25 per vial) containing either 0.4% agar/PBS (starved) or standard *Drosophila* medium (control).

### Intestinal dissection and microscopy

Adult female flies were surface sterilized by rinsing with 75% ethanol in a Petri dish. Flies were then transferred to a watch glass containing ice-cold 1×PBS for dissection under a light microscope, where intestines were isolated using fine forceps. Dissected intestines were fixed in 4% paraformaldehyde (Electron Microscopy Sciences) in 1×PBS for 20 min at room temperature. Tissues were then stained with Hoechst 33342 dye (1:1000 dilution) for 10 min, followed by three 15-min washes in 1×PBS. For mounting, tissues were placed on glass slides with coverslips using Vectashield mounting medium (Vector Laboratories Inc.). Slides were visualized using a Zeiss LSM 880 laser confocal microscope with 10× and 20× objectives and Axiovision software.

### qRT-PCR

For whole-body samples, flies were transferred into 1.5 ml microtubes in groups of five before snap freezing on dry ice. For tissue samples, groups of five to ten female tissues were dissected in ice-cold 1×PBS, and tissues were immediately transferred to 1.5 ml microtubes containing 500 μl TRIzol reagent (Invitrogen) before snap freezing on dry ice. For abdominal tissue samples, guts and ovaries were removed prior to RNA extraction. Total RNA was extracted from either whole flies, whole larvae or isolated adult tissues according to manufacturer's instructions (Invitrogen; 15596-018). RNA samples were DNase treated following manufacturer's instructions (Ambion; 2238 G). Reverse transcription was achieved using SuperScript III (Invitrogen; 18080044). The generated cDNA was used as a template for qRT-PCR reactions (QuantStudio 6 RT-PCR system, Applied Biosystems) using the primer pairs defined in Table S2 and SYBR Green reagents (Applied Biosystems, Foster City, CA, USA). Data analysis for qRT-PCR was completed using the comparative C_T_ method (2^−ΔΔCT^). PCR data were normalized to reference genes for which expression levels were unaffected by the experimental conditions.

### Statistics

For all experiments, error bars represent s.e.m., and *P*-values are the result of unpaired two-tailed *t*-test or two-way ANOVA followed by Student's *t*-test using GraphPad Prism (v.9). *P*<0.05 was considered statistically significant.

## Supplementary Material

10.1242/dmm.052999_sup1 Supplementary information
