# Supplementary material for: Gut-derived Unpaired-3 cytokine signaling promotes systemic hypoxia tolerance in Drosophila
Source: Dis Model Mech. 2026 Sep 1;19(8):dmm052999. doi: 10.1242/dmm.052999 (PMC13580592; doi:10.1242/dmm.052999)
Supplement: Supplementary file 1 [file dmm-19-052999-s1.pdf]

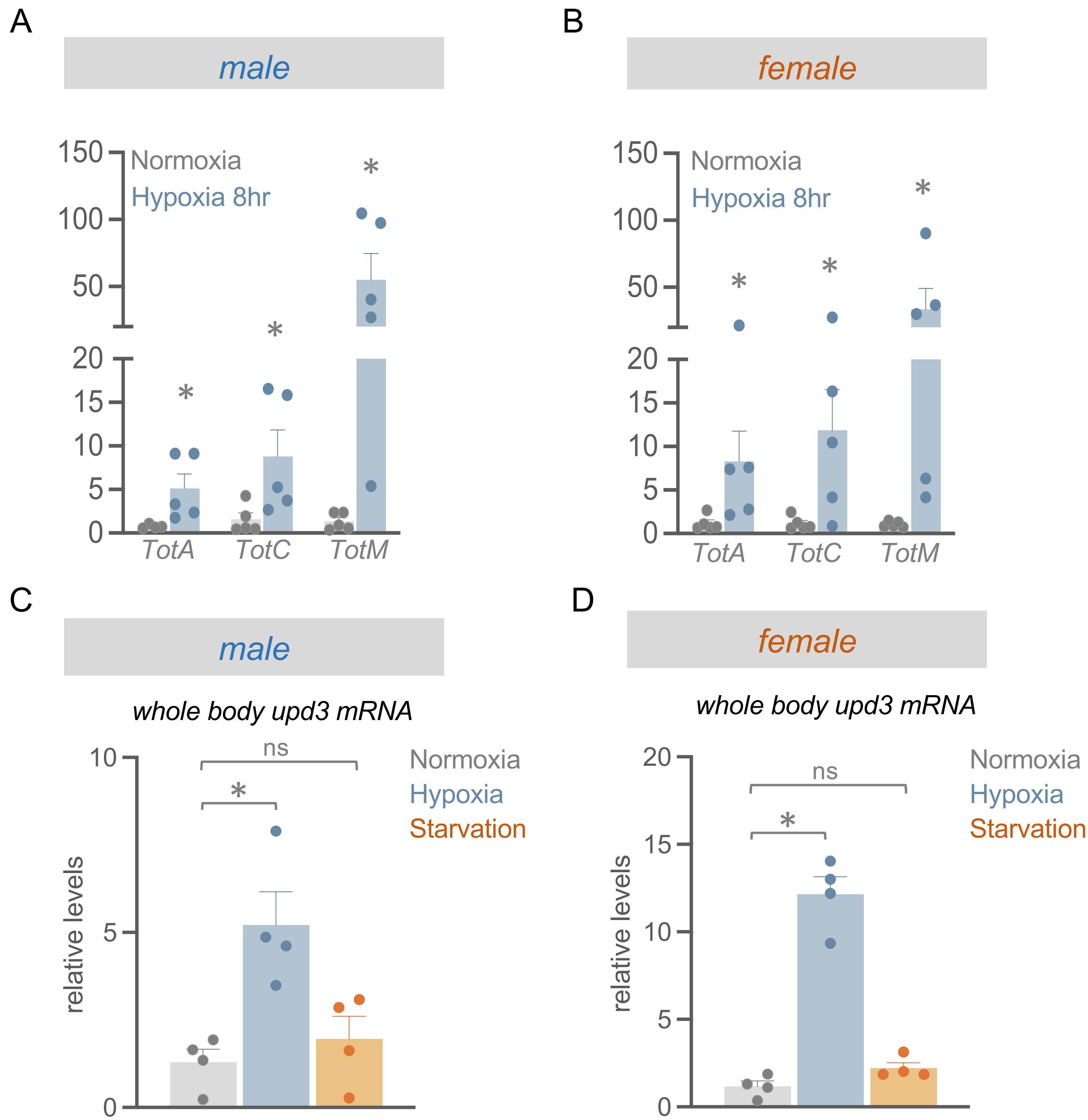

Fig. S1. Starvation does not induce upd3 expression.

A-B) *w*[1118] adult male and female flies were maintained in normoxia or exposed to 8 hours of hypoxia, then STAT-dependent *Turandot* genes were profiled. *TotA, TotC and TotM* were induced in both A) male and B) female adults. All qRT-PCR data were normalized to reference genes listed in Table S2. Data represents mean + SEM, N=4 independent groups of samples (5 animals per group) per experimental condition. Data points represent independent samples. *p < 0.05, compared to normoxia controls.

C-D) *w*[1118] adult male and female flies were maintained in normoxia, exposed to 16 hours of hypoxia or 16 hours starvation. Upd3 expression in whole animals was measured by qRT-PCR. Upon starvation, there is no significant change in *upd3* mRNA levels in either C) male or D) female adults. All qRT-PCR data were normalized to reference genes listed in Table S2. Data represents mean + SEM, N=4 independent groups of samples (5 animals per group) per experimental condition. *p < 0.05, ns not significant, unpaired *t*-test. Data points represent independent samples.

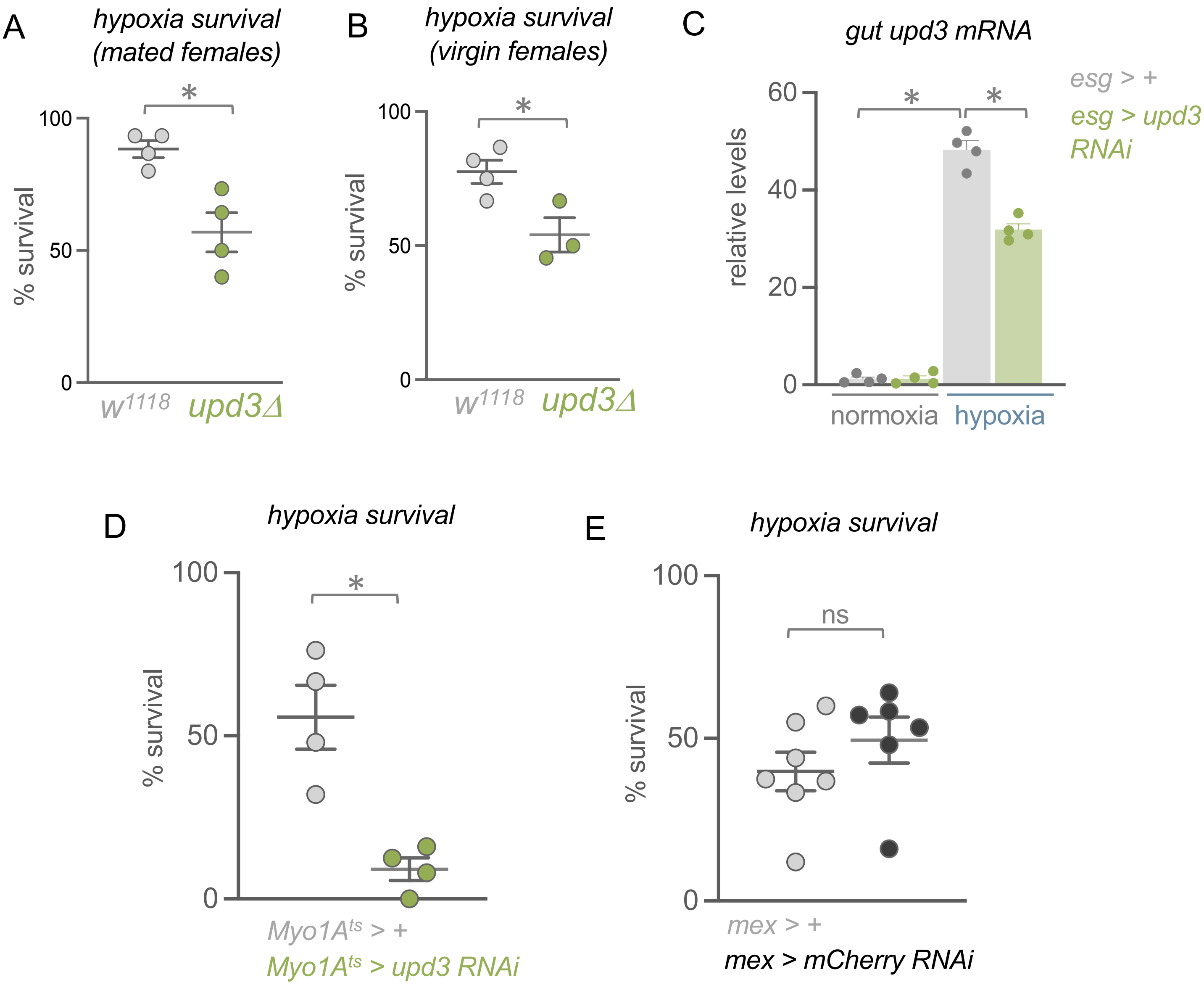

Fig. S2. Female gut upd3 knockdown reduces hypoxia survival. A, B) Age-matched mated and virgin control (w1118) and upd3-null (upd3Δ) female flies were exposed to hypoxia and survival was measured. The requirement for upd3 in hypoxia survival was not dependent on mating status in female flies – upd3-null (upd3Δ) A) mated and B) virgin females both showed reduced survival compared to mated or virgin controls (w1118). C) qRT-PCR measurement of upd3 mRNA in whole intestines from control (esg > +) vs progenitor cell (intestinal stem cells + enteroblasts) upd3 knockdown flies (esg > upd3-RNAi) in hypoxia. All qRT-PCR data were normalized to reference genes listed in Table S2. Data represents mean + SEM, N=4 independent groups of samples (5 intestines per group) per experimental condition. *p < 0.05, Student's t-test following 2-way ANOVA. Data points represent independent samples. D) Hypoxia survival of control (Myo1Ats > +) vs gut upd3 RNAi (Myo1Ats > upd3-RNAi) adult female flies. Data represents mean ± SEM, N=4 independent groups of animals (15-25 per group) per experimental condition. *p < 0.05, unpaired t-test. Data points represent individual samples. E) Hypoxia survival measured in control (mex > +) vs enterocyte mCherry RNAi (mex > mCherry RNAi) animals. Data represents mean ± SEM, N≥6 independent groups of animals (15-25 per group) per experimental condition. *p < 0.05, unpaired t-test. Data points represent individual samples.

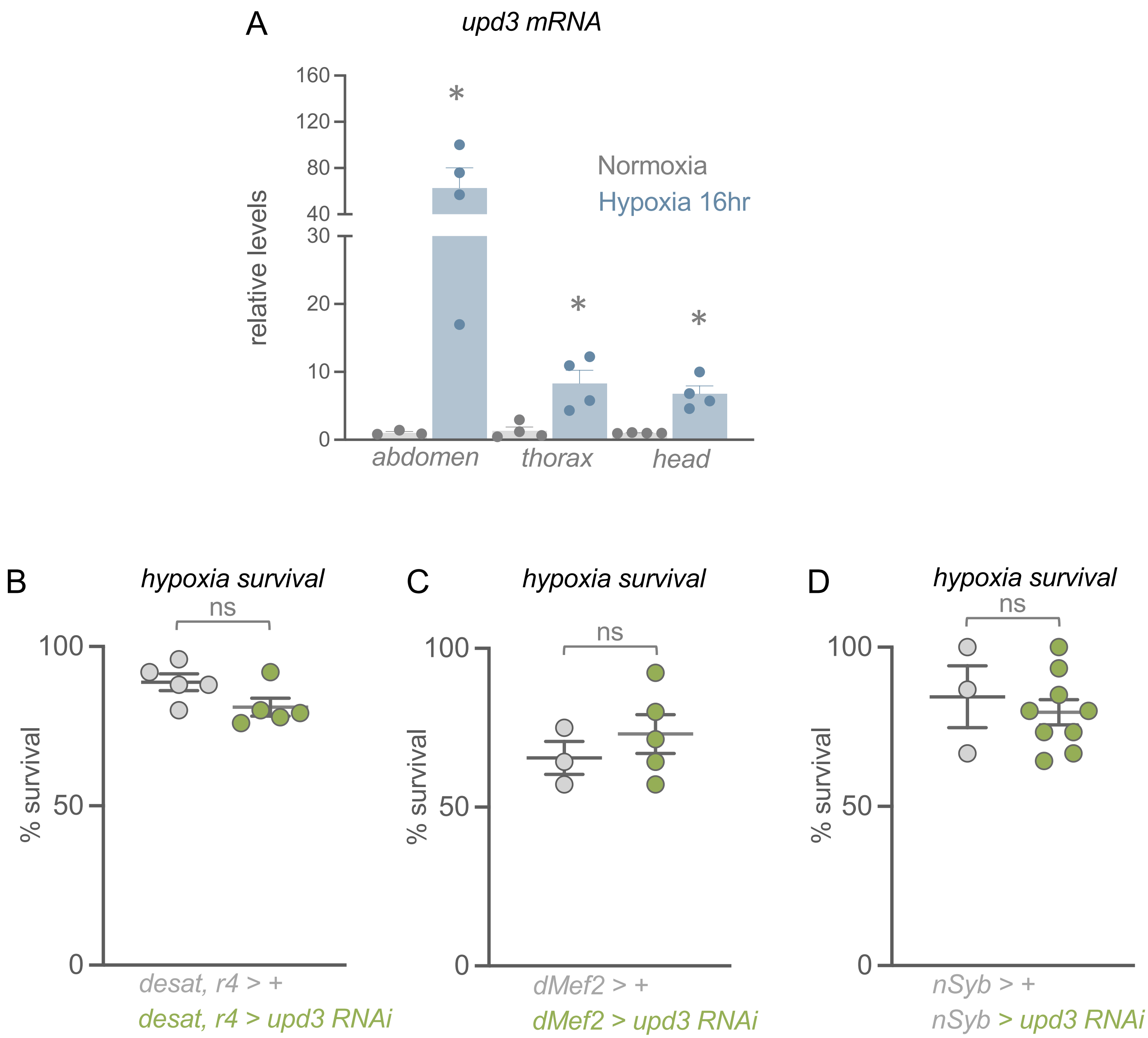

Fig. S3. Tissue-specific upd3 roles in hypoxia tolerance

A) qRT-PCR analysis of upd3 mRNA levels from isolated abdomens (with intestine and ovaries removed), thorax and heads of w1118 adult females maintained in normoxia or exposed to 16 hours of hypoxia (1% oxygen). All qRT-PCR data were normalized to reference genes listed in Table S2. Data represents mean + SEM, N=4 independent groups of samples (5 animals per group) per experimental condition. Data points represent independent samples. *p < 0.05 compared to normoxia controls.

B-D) Hypoxia survival of control female flies (Desat, r4 > +) vs flies with RNAi-mediated knockdown of upd3 in B) fat body and oenocytes combined (Desat, r4 > upd3 RNAi), C) muscle (dMef2 > upd3 RNAi), or D) neurons (nSyb > upd3 RNAi). Data represents mean ± SEM. Data points represent individual groups (15-25 per group). ns, p > 0.05, unpaired t-test.

Disease Models & Mechanisms • Supplementary information

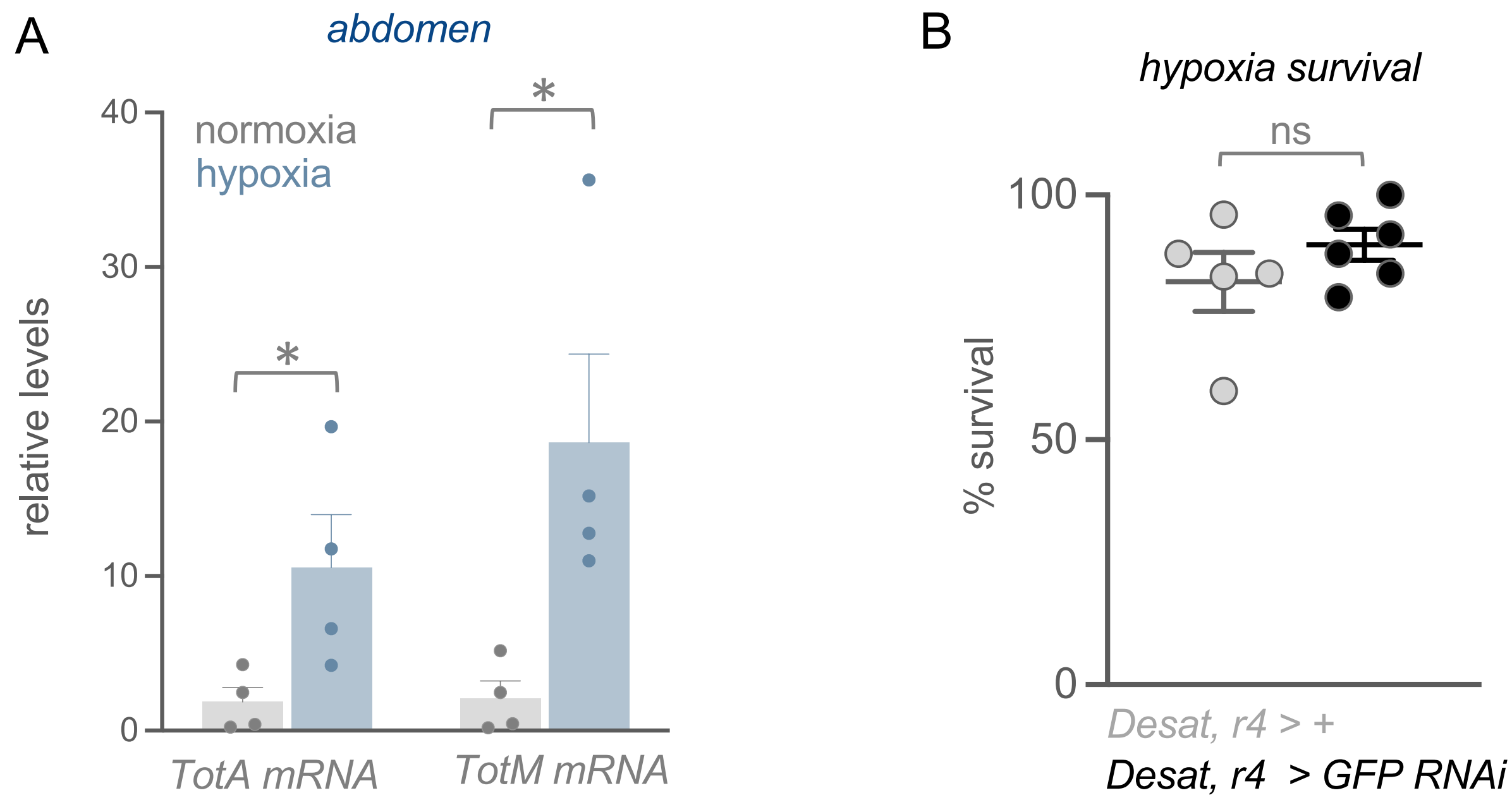

Fig. S4. Abdominal tissue induction of JAK/STAT signaling in hypoxia.

A) qRT-PCR analysis of STAT target genes (Tot A and Tot M) in the adult female abdominal tissue from flies maintained in normoxia or exposed to hypoxia for 16 hrs. All qRT-PCR data were normalized to reference genes listed in Table S2. Bars represent mean + S.E.M. Data points represent independent samples. *$p < 0.05$, unpaired t-test.

B) Hypoxia survival of control female flies (Gal4 driver > +) vs flies with GFP RNAi targeted to fat body and oenocytes combined (Desat, r4 > GFP RNAi). Data represents mean ± SEM. Data points represent individual groups (15-25 per group). ns, $p > 0.05$, unpaired t-test

Disease Models & Mechanisms • Supplementary information

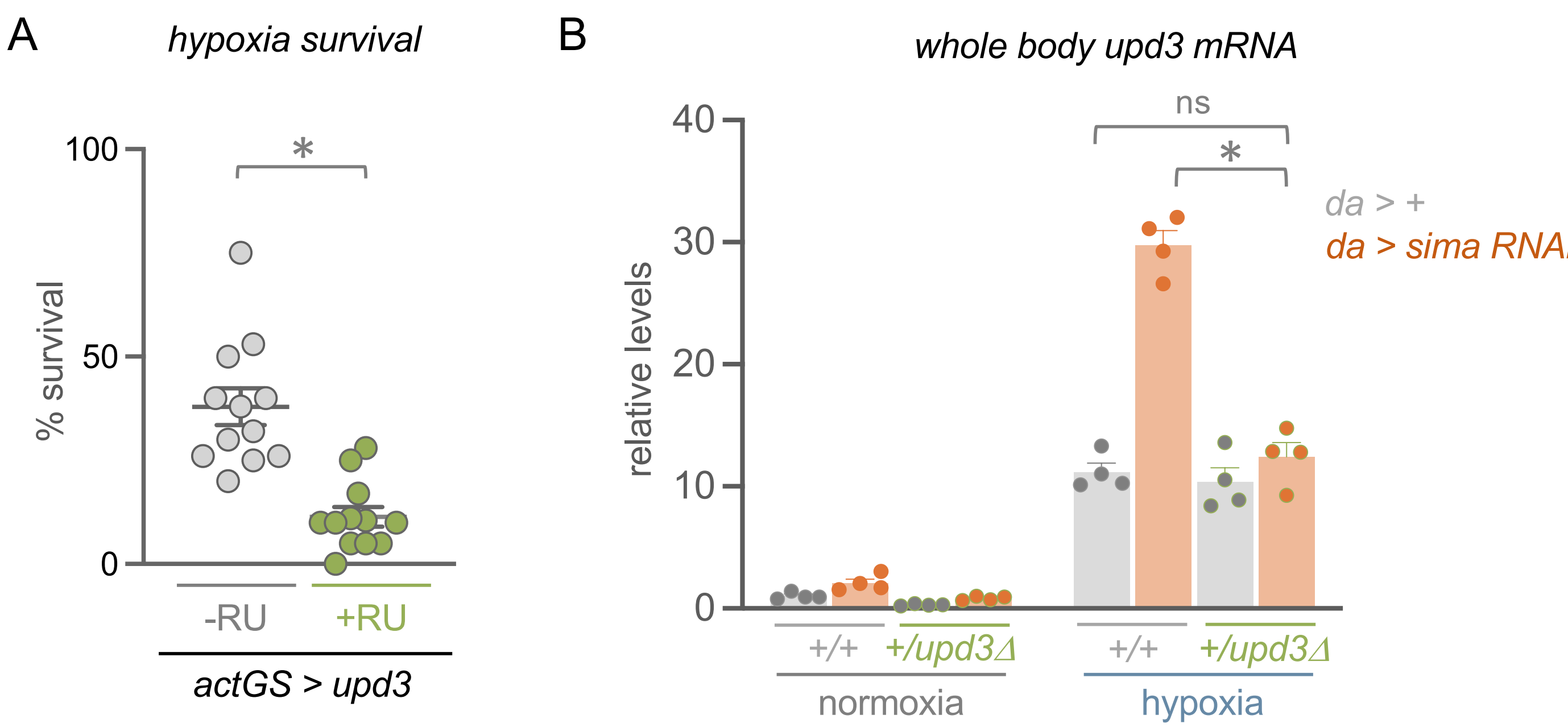

Fig. S5. Sima-dependent control of upd3

A) Hypoxia survival of control (*act-GS > upd3*, -RU486) vs *upd3* OE (*act-GS > upd3*, +RU486) adult flies. Data represents mean ± SEM, N=12 independent groups of animals (15-25 per group) per experimental condition. *p < 0.05, unpaired *t*-test. Data points represent individual samples.

B) Control *(da > +)*, *upd3*Δ heterozygote (*da > upd3*Δ/+), *sima* knockdown (*da>sima-RNAi*) and *upd3*Δ heterozygote *sima* knockdown (*da > upd3*Δ*/+;sima-RNAi*) adult females were maintained in normoxia or exposed to 16h hypoxia. Hyper-induction of *upd3* mRNA levels seen in *sima* knockdown animals is abrogated in an upd3 heterozygote (*upd3*Δ/+) background. All qRT-PCR data were normalized to reference genes listed in Table S2. Data represents mean + SEM, N=4 independent groups of samples (5 animals per group) per experimental condition. *p < 0.05, ns not significant, Student's *t*-test following 2-way ANOVA. Data points represent independent samples.

Disease Models & Mechanisms • Supplementary information

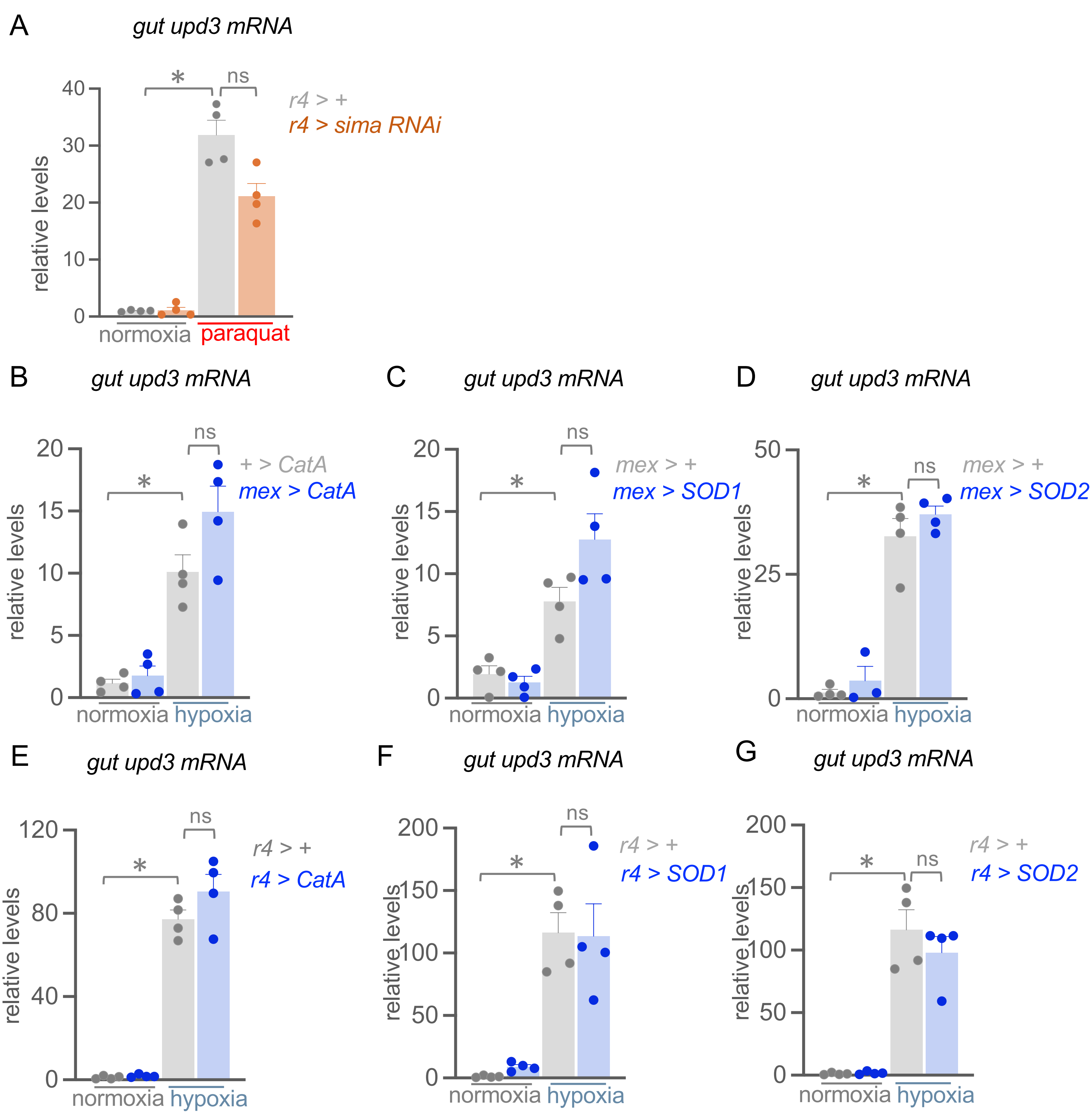

Fig. S6. Reactive oxygen species and the control of intestinal upd3 levels. A) Fat body sima knockdown (*r4 > sima-RNAi*) did not affect intestinal upd3 levels compared to controls (*r4* > +) when animals were fed oxidative stress inducing chemical paraquat. All qRT-PCR data were normalized to reference genes listed in Table S2. Data represents mean + SEM, N=4 independent groups of samples (5 intestines per group) per experimental condition. *p < 0.05, ns not significant, Student's *t*-test following 2-way ANOVA. Data points represent independent samples. B-D) Intestinal overexpression of B) ROS scavenger Catalase A (*mex > CatA*), C) SOD1 (*mex > SOD1*), or D) SOD2 (*mex* > SOD2) failed to block intestinal induction of *upd3* compared to controls in hypoxia. All qRT-PCR data were normalized to reference genes listed in Table S2. Data represents mean + SEM, N=4 independent groups of samples (5 intestines per group) per experimental condition. *p < 0.05, ns not significant, Student's *t*-test following 2-way ANOVA. Data points represent independent samples. E-G) Fat body overexpression of E) ROS scavenger Catalase A (*r4 > CatA*), F) SOD1 (*r4 > SOD1*), or G) SOD2 (*r4 > SOD2*) failed to block intestinal induction of *upd3* compared to controls in hypoxia. Data represents mean + SEM, N=4 independent groups of samples (5 intestines per group) per experimental condition. *p < 0.05, ns not significant, Student's *t*-test following 2-way ANOVA. Data points represent independent samples.

Table S1. *Drosophila* stocks used in this work.

Name	Genotype	Source
upd3Gal4,UAS-GFP	+; upd3-gal4, UAS-GFP/Cyo; +	Gifted from Erica Bach
upd3Δ	w, upd3Δ; +; +	BDSC (55728)
UAS-upd3	w^{1118}; UAS-upd3; +	Gifted from Bruno Lemaitre
UAS-upd3 RNAi	+; +; UAS-upd3 RNAi	VDRC (27136/GD)
UAS-sima RNAi	+; UAS-sima RNAi; +	VDRC (106187/KK)
UAS-NOS RNAi	yv; UAS-NOS RNAi; +	BDSC (57700)
UAS-STAT92E RNAi	+; UAS-STAT92E RNAi; +	VDRC (43866/GD)
UAS-CatA	w; UAS-CatA; +	BDSC (24621)
UAS-SOD1	w; UAS-SOD1	BDSC (33605)
UAS-SOD2	w; UAS-SOD2	BDSC (24494)
UAS-Bnl RNAi	W; UAS-bnl RNAi	VDRC (101377)
UAS-Hipk RNAi	yv; +; UAS-Hipk RNAi;	BDSC (35363)
mexGal4^{ts}	mex-GAL4/CyO; tubgal80ts, UAS-nlsGFP/TM6B	Gifted from Bruce Edgar
da-Gal4	w^{1118}; +; da-gal4	BDSC (8641)
r4-Gal4	w^{1118}; +; r4-gal4	BDSC (33832)
esg-Gal4	W^{1118}esg-Gal4	Gifted from Bruce Edgar
nSyb-Gal4	yw; nSyb-Gal4	BDSC (51941)
dMef2-Gal4	W; dMef2-Gal4	BDSC (25756)
Desat-Gal4	w; desatGal4/Cyo; +	BDSC (65404)
mex-Gal4	w^{1118}; mex-gal4; +	BDSC (91368)
Myo1A-Gal4	w; Myo1A-gal4; +	Gifted from Bruce Edgar
Myo1A-Gal4^{ts}	w; Myo1AGal4 tubGal80ts UAS-GFP	Jiang et al., 2009
da-GS	w^{1118}; da-gal4 GeneSwitch; +	Sun et al (2014)
Act-GS	w^{1118}; Act-gal4 GeneSwitch; +	Sun et al (2014)
UAS-GFP RNAi	W1118; UAS-GFP-RNAi	BDSC (9330)
UAS-mCherry RNAi	yv; + ; UAS-mCherry RNAi	BDSC (35785)

Disease Models & Mechanisms • Supplementary information

Table S2. Primers used for qRT-PCR.

All primers listed in 5’-3’ direction. Reference genes used in this study are indicated by an asterisk (*).

Gene	Forward	Reverse
Upd3	ACAAGGCCAGGATCACCACCAAT	TGTACAGCAGGTTGGTCAGGTTGA
Socs36e	AGTGCTTTACTGCTGCGACT	TCGTCGAGTATTGCGAAGT
TotA	TCAACTGCTCTTATGTGCTTTGC	CTCACGATCTTCGTCGGAATAG
TotC	GCCTCCATTTCTCTACTATGCC	TCCCTTTCCTCGTCAGAATAGC
TotM	TTGAGCTGCCTTATGGTCTTCT	CGCTGTTTTTCTGTGACGAACT
Nos	ATGTCGCAGCATTTCACATCG	GGCGTTGCTTGAGTTTTGATTT
bnl	TGCCCTATCACAGAGTTGC	ACCTACACGAACGCCATCAC
Hipk	CAACAATGTCAAGGCATC	CAGGCTGCACAGTGTGGAAA
**5S rRNA*	ACGACCATACCACGCTGAAT	AGCGGTCCCCCATCTAAGTA
**18S rRNA*	ATGCACCACCACCCATAGAT	CCTGCGGCTTAATTTGACTC
**eIF-2α*	TCTTCGATGAGTGCAACCTG	CCTCGTAACCGTAGCAGGAG
**RpS24*	CCATGTACAAGGTCACCCC	CGGTACTTGGGCTCGAACTT

Disease Models & Mechanisms • Supplementary information
